# *Cnih3* Deletion Dysregulates Dorsal Hippocampal Transcription across the Estrous Cycle

**DOI:** 10.1523/ENEURO.0153-22.2023

**Published:** 2023-03-13

**Authors:** Bernard Mulvey, Hannah E. Frye, Tania Lintz, Stuart Fass, Eric Tycksen, Elliot C. Nelson, Jose A. Morón, Joseph D. Dougherty

**Affiliations:** 1 Department of Genetics, Washington University School of Medicine, St. Louis, MO 63110; 2 Department of Psychiatry, Washington University School of Medicine, St. Louis, MO 63110; 3 Department of Anesthesiology, Washington University School of Medicine, St. Louis, MO 63110; 4 McDonnell Genome Institute, Washington University School of Medicine, St. Louis, MO 63110

**Keywords:** candidate gene, gene expression, opioid use disorder, sex differences, sex hormones

## Abstract

In females, the hippocampus, a critical brain region for coordination of learning, memory, and behavior, displays altered physiology and behavioral output across the estrous or menstrual cycle. However, the molecular effectors and cell types underlying these observed cyclic changes have only been partially characterized to date. Recently, profiling of mice null for the AMPA receptor trafficking gene *Cnih3* have demonstrated estrous-dependent phenotypes in dorsal hippocampal synaptic plasticity, composition, and learning/memory. We therefore profiled dorsal hippocampal transcriptomes from female mice in each estrous cycle stage, and contrasted it with that of males, across wild-type (WT) and *Cnih3* mutants. In wild types, we identified only subtle differences in gene expression between the sexes, while comparing estrous stages to one another revealed up to >1000 differentially expressed genes (DEGs). These estrous-responsive genes are especially enriched in gene markers of oligodendrocytes and the dentate gyrus, and in functional gene sets relating to estrogen response, potassium channels, and synaptic gene splicing. Surprisingly, *Cnih3* knock-outs (KOs) showed far broader transcriptomic differences between estrous cycle stages and males. Moreover, *Cnih3* knock-out drove subtle but extensive expression changes accentuating sex differential expression at diestrus and estrus. Altogether, our profiling highlights cell types and molecular systems potentially impacted by estrous-specific gene expression patterns in the adult dorsal hippocampus, enabling mechanistic hypothesis generation for future studies of sex-differential neuropsychiatric function and dysfunction. Moreover, these findings suggest an unrecognized role of *Cnih3* in buffering against transcriptional effects of estrous, providing a candidate molecular mechanism to explain estrous-dependent phenotypes observed with *Cnih3* loss.

## Significance Statement

*Cnih3* mutants show estrous-dependent alterations in learning, as well as physiological and anatomic changes in the dorsal hippocampus. However, the transcriptomic consequences of the estrous cycle on gene expression in the dorsal hippocampus of mice, including of *Cnih3* mutants, have not been characterized. Here, we identify candidate cell types, pathways, and gene regulators putatively involved in estrous-dependent gene expression in wild-type (WT) mice. We then contrast these with dorsal hippocampal transcriptomics in *Cnih3* knock-out (KO) mice. Using our wild-type data as a reference, we demonstrate that *Cnih3* knock-out mice have accentuated transcriptional responses across the estrous cycle.

## Introduction

Neurobehavioral sex differences are well-established factors in physiologic and pathologic processes ranging from reproduction and parenting to depression and addiction, in part through acute “activational” effects of sex hormones (Arnold, 2009). Example activational effects include regulation of dopamine turnover, suppression of GABA signaling (Del Río et al., 2018), and estrogenic stimulation of corticosteroid release through hypothalamic-pituitary-adrenal (HPA) axis upregulation of *CRH* (in contrast to its repression by androgens; Lund et al., 2004; Bao et al., 2006). Perhaps unsurprisingly, then, the human menstrual and rodent estrous cycles continually alter the microstructure of the brain: sex-specific responses to social stress in rat medial prefrontal cortex have implicated estrous-dependent alterations to cellular communication (Duclot and Kabbaj, 2015).

Phenotypically, the impact of sex has been observed in neuropsychiatric diseases and rodent models thereof (Kuhn, 2015), partly by way of the hippocampus in addiction (Kohtz and Aston-Jones, 2016). Hippocampal synaptic density peaks during proestrus, when estradiol and progesterone are highest (Woolley and McEwen, 1992), affecting learning and memory (Frick et al., 2015; Frick and Kim, 2018). In the mouse hippocampus, this increase in synaptic density is accompanied by elevated expression of synaptic transmission genes during proestrus and diestrus in comparison to males (Jaric et al., 2019). A longitudinal human study across the menstrual cycle revealed hippocampal changes suggestive of increased myelination during high estrogen periods (Barth et al., 2016), paralleling changes seen in rodent models during short-term learning. Despite such physiologic and behavioral observations, however, characterization of their potential molecular mediators has been less intensive to date.

The dorsal hippocampus, an interface of stress, sex, and drug-seeking behavior (Henderson et al., 2015; Johnson et al., 2021), is essential for drug-induced associative learning (Fakira et al., 2016). Consistent with this, mouse knock-out (KO) of the opioid dependence-associated gene *Cnih3* (Nelson et al., 2016) results in not only sex- but estrous-stage-specific effects on learning and memory (H.E. Frye et al., 2021). *Cnih3* is an AMPA receptor (AMPAR) trafficking protein that delivers AMPARs to the postsynaptic membrane, enhancing their activity (Schwenk et al., 2009; Coombs et al., 2012; Herring et al., 2013; Shanks et al., 2014; Brown et al., 2018). Hippocampal AMPARs have a clear role in learning and memory (Lee et al., 2003; Kessels and Malinow, 2009); accordingly, *Cnih3* KO showed severe memory deficits, altered biochemical localization of AMPARs, and corresponding synaptic changes, *but* only in female mice, and limited to particular phases of the estrous cycle (H.E. Frye et al., 2021). This suggested the surprising hypothesis that *Cnih3* is involved in buffering the female brain against cyclic changes in hippocampal learning.

A recent synthesis across several studies of wild-type (WT) mouse hippocampus found that very few genes are consistently called sex DE (Ocañas et al., 2022). The granular manner in which the estrous cycle alters brain gene expression and pathways, even in WT rodents, has only been characterized in limited phases (Iqbal et al., 2020) or not yet in direct contrast to males (DiCarlo et al., 2017). Therefore, we first sought to determine how extensively WT dorsal hippocampal gene expression fluctuates across estrous, as grouping all females together may underlie the reported paucity of sex DEGs via unaccounted-for biological variance and/or net male-equivocal expression. Given recent evidence of dorsal hippocampal *Cnih3* expression and cycle-dependent cellular and plasticity changes with *Cnih3 loss* (H.E. Frye et al., 2021), we then sought to transcriptionally characterize the mouse dorsal hippocampus at each stage of the estrous cycle in *Cnih3* KO mice to clarify the molecular effects of KO.

In WTs, we only identify male-female divergence of autosomal gene expression when considering individual estrous stages. Using gene pattern analysis, we define cyclic expression patterns of the female dorsal hippocampus and these patterns’ enrichment in cell type markers, related transcriptomic signatures, and candidate regulators, providing an extensive resource for hypothesis generation in the study of interplay between estrous and dorsal hippocampal function. We then examined differences between *Cnih3* KO males and KO females in specific phases of estrous and identified a profound enhancement in gene expression differences, resulting in substantially more differential genes between sexes.

Finally, we examined expression differences between WT versus KO altogether, in males and in each estrous stage. These analyses, when compared with those within KOs or WTs, suggested that subtle changes were being induced by *Cnih3* KO to accentuate sex-differential expression. Indeed, we observed that the magnitude of sex-differential expression at diestrus or estrus was greater in the KO regardless of false discovery rate (FDR) level, supporting the hypothesis that *Cnih3* buffers against excess gene-regulatory responses to cycling sex hormones.

## Materials and Methods

### Animals, estrous staging, and dorsal hippocampal dissections

Procedures were approved by the Institutional Animal Care and Use Committee at Washington University in St. Louis. Adult (age range 12–24 weeks; see Extended Data Table 1-1) WT and *Cnih3* KO littermates on a C57/BL6j background (>10 backcrossed generations) were used. This *Cnih3* KO mouse line was previously generated beginning in 2015 from *Cnih3^tm1a(KOMP)Wtsi^* BL/6N mice (Knockout Mouse Project); full details of the KO line were previously published (H.E. Frye et al., 2021). Briefly, the KO line lacks exon four of *Cnih3*, frameshifting exons 5 and 6 and thus truncating the CNIH3 protein. We confirmed the previously reported loss of exon 4 in our experimental animals by examining alignment of RNA-sequencing (RNA-seq) reads within the *Cnih3* transcript (Extended Data Figs. 4-1, 4-2). Genotyping was performed according to methods used by the group publishing the mouse line (H.E. Frye et al., 2021). *Cnih3* heterozygotes were mated in pairs (or occasionally to *Cnih3* homozygotes) to generate homozygous KO offspring for RNA-seq. Because of the large number of mice required to cover all estrous cycle stages in two genotypes in the midst of pandemic-era animal facility constraints, mice we subsequently refer to as WT were collected from a mix of offspring from WT B6 x WT B6 matings and *Cnih3* mutant-negative littermates from *Cnih3* heterozygote x *Cnih3* heterozygote matings (parental genotypes given in Extended Data Table 1-1). Principal component analysis (PCA; Extended Data Fig. 1-2) did not identify a clear axis of variation relating to parental genotype in the WT animals; thus, we collapsed all wild-type animals together in a single group, regardless of parent genotype.

Mice were kept in climate-controlled facilities with a 12/12 h light/dark cycle and *ad libitum* access to food and water. The estrous cycle was monitored by vaginal lavage of sterile saline for at least two consecutive days before tissue harvesting. Samples were allowed to dry on glass slides, rinsed with water, and stained with giemsa stain (Ricca Chemical) to improve contrast and differentiate between cell types. Vaginal cell cytological analysis was used to identify estrous cycle stages by three independent observers. Estrus (E, Est) was characterized by the presence of cornified epithelial cells, metestrus (M, Met) by a mix of leukocytes and both nucleated and cornified epithelial cells, diestrus (D, Di) by leukocytes, and proestrus (P, Pro) by nucleated epithelial cells. Males and females at each stage of the estrous cycle (*n* = 4–7 per group) were decapitated and dorsal hippocampi were rapidly dissected over ice by a single researcher in the afternoon using methods previously described (Xia et al., 2011; Portugal et al., 2014; Fakira et al., 2016), and tissue was snap frozen on dry ice and stored at −80°C until use. Besides estrous staging before decapitation, mice used in this study were not used for anything other than the RNAseq analysis. In order to ensure responsible and judicious use of animal specimens, additional brain regions and samples were banked for potential future use in other studies.

### Tissue processing and RNA purification

The dorsal-most 1/3 of each hippocampus was collected for RNA preparation. Hippocampi were placed in 500 μl of buffer [50 mm Tris, pH 7.4, 100 mm NaCl, 1% NP-40, supplemented with Rnasin (Promega) and protease inhibitors (Roche)] on ice. Samples were homogenized in buffer solution on ice using a handheld motorized pellet pestle, and lysate was then centrifuged at 2000 × *g* for 15 min at 4°C, and 133 μl of the supernatant was taken for RNA purification. This was mixed with 67 μl of Promega’s simplyRNA Tissue kit Homogenization buffer with 1-thioglycerol (20 μl/ml), then 200 μl of the Promega kit’s lysis buffer, and extracted using a Maxwell RSC 48 robot (Promega) following the manufacturer’s instructions.

### RNAseq library preparation and sequencing

Total RNA integrity was determined using Agilent Bioanalyzer or 4200 Tapestation. Library preparation was performed with 10 ng of total RNA for samples with a Bioanalyzer RIN score >8.0. The resulting Poly-A enriched, double-stranded cDNA (dscDNA) was prepared using the SMARTer Ultra Low RNA kit for Illumina Sequencing (Takara-Clontech) per manufacturer’s protocol. cDNA was fragmented using a Covaris E220 sonicator using peak incident power 18, duty factor 20%, cycles per burst 50 for 120 s. cDNA was blunt ended, had an A base added to the 3′ ends, and then had Illumina sequencing adapters ligated to the ends. Ligated fragments were then amplified for 14 cycles using primers incorporating unique dual index tags according to manufacturer’s protocol. Fragments were sequenced on an Illumina NovaSeq-6000 using paired end reads extending 150 bases.

### Quantitative PCR and analysis

Using the same RNA as collected above, we synthesized single-stranded cDNA from 125 ng of each sample RNA using the qScript Reverse Transcriptase kit (QuantaBio #95047) per manufacturer instructions in 20-μl reactions. Six of these RNA samples were also prepared in a second reaction with 125 ng input RNA but no reverse transcriptase enzyme as a negative control for the quantitative PCR (qPCR) reactions. A total of 200-μl water was added to each reaction after completion, and 4 μl of the diluted reaction used as template for each qPCR reaction well. A total of 384-well plates were prepared with technical triplicate reactions (i.e., three reactions with 4 μl cDNA each) per sample for each of two genes: one gene of interest, and β-actin as within-sample normalization controls; 6 μl of a mastermix comprised of 5 μl per reaction of PowerUp SyBr Green 2× Mastermix (Applied Biosciences #A25742) and 0.5 μl per reaction of each primer at 10 μm (final concentration 500 nm) was added to each well of cDNA for a total of 10 μl. The primers used were: β-actin forward, 5′-AGAGGGAAATCGTGCGTGAC-3′; β-actin reverse, 5′-CAATAGTGATGACCTGGCCGT-3′; *Otx2* forward, 5′-GAATCCAGGGTGCAGGTATGG-3′; *Otx2* reverse, 5′-CTGAACTCACTTCCCGAGCTG-3′; *Prlr* forward, 5′-CTGCACTTGCTTACATGCTGC-3′; *Prlr* reverse, 5′-GGGGAACGACATTTGTGGATTTC-3′; *Prl* forward, 5′-CCAATCTGTTCCGCTGGTGA-3′; and *Prl* reverse, 5′-GGGACTTTCAGGGCTTGTTCC-3′. A 3 min 95°C hot-start step was followed by 40 cycles of 95°C for 15 s and 63°C (annealing + elongation) for 20 s. Immediately after, a 95°C hold for 15 s and a temperature ramp from 60°C to 95°C were executed for melt curve analysis. Cycling, threshold detection, and melt curve analysis for each plate was performed on a Quantstudio 6 instrument. Wells without amplification, with melt curve peaks under 80°C, or diverging ≥1 cycle to threshold of detection (CT) from the other two technical replicates for a sample were discarded from analysis if the other two wells strongly reflected one another. Delta CT (dCT) between the target gene and actin was calculated for each technical replicate of the target gene within a sample by subtracting that sample’s mean actin CT. These repeated measurements were used as input for plotting as boxplots in Extended Data Figure 2-4 and used in a repeated-measures ANOVA to test each pairwise comparison of interest as defined by the RNA-seq data. Twelve total comparisons of interest were investigated across the three target genes. In all cases, no-reverse-transcriptase negative controls produced either no products, only primer dimers, or the target product but at >5 CT later than any reverse transcribed sample on the plate.

### Data analysis

RNA-seq reads were aligned to the Ensembl release 101 primary assembly (GRCm38.101) with STAR version 2.7.9a (Dobin et al., 2013). Gene counts were derived from the number of uniquely aligned unambiguous reads by Subread:featureCount version 2.0.3. All gene counts were then imported into the R/Bioconductor package EdgeR (Robinson et al., 2010) and TMM normalization size factors were calculated to adjust for samples for differences in library size. Ribosomal genes and genes not expressed in at least ten samples greater than one count-per-million were excluded from further analysis. The TMM size factors and the matrix of counts were then imported into the R/Bioconductor package Limma (Ritchie et al., 2015). Weighted likelihoods based on the observed mean-variance relationship of every gene and sample (i.e., one model for all 10 groups, each estrous stage/males and each genotype) were then calculated for all samples and the count matrix was transformed to moderated log2 counts-per-million with Limma’s voomWithQualityWeights (Liu et al., 2015). Differential expression analysis was then performed to analyze for differences between pairs of conditions; results were filtered to genes with Benjamini–Hochberg FDR adjusted *p*-values less than or equal to 0.05 except where noted. All differential expression analyses used a single input dataset covering all WT and *Cnih3* KO samples and model using a Limma contrast matrix to avoid the need for *post hoc* multiple testing corrections: *expression ∼ 0 + group* (where group = genotype and estrous stage or male). Estrous-stage agnostic and sex-agnostic (global genotype effect) contrasts were collapsed into aggregated contrast coefficients for each stage together; for example, WT female versus WT male DE = (0.25*metestrus + 0.25*diestrus …) – male.

Using the ssizeRNA package in R, we estimated that our experiment as executed was powered with β = 0.862 for an FDR threshold of 0.05 with a sample size of 6 per group, 5% of genes as DE at |FC| of ≥±2, mean count of 3000 (based on the analyzed counts table), and the global dispersion, which by nature will be influenced by age variability across the cohort, of the analyzed counts (0.048).

Because of the number of mice and conditions to be collected, ages were not ideally balanced in the dataset. As brain gene expression differences in B6 lineage mice from ages one to four months have been observed (Bundy et al., 2017), we investigated whether age had a substantial impact on our results. We used the R package ComBat-seq (Zhang et al., 2020) to remove age effects (with age as categorical effect in months) while preserving genotype-stage/sex effects before a parallel, exploratory limma analysis. This two-step procedure constituted first correcting the library-size-corrected counts for age using ComBat-seq, followed by the same limma-voom steps as used in the original analysis (see below, Code availability). 3D principal component analysis (PCA) was used to compare the age-adjusted data to the original analysis (Extended Data Fig. 1-1).

For gene pattern analysis, the degPatterns function of R package DEGreports package (Pantano et al., 2022) was used with the moderated log2 counts per million (CPM) expression values for an input gene set to examine the patterns of expression over each stage/condition examined. The degPatterns parameters used were as follows: minimum number of genes fitting a pattern to report the pattern is 5 (*minc *=* *5) and outliers were excluded from final clusters (*reduce* = TRUE).

For ontology, cell type, and regulatory enrichments, the Enrichr web tool (E.Y. Chen et al., 2013) was used, entering the list of significant differentially expressed genes (DEGs; at FDR < 0.05) from the differential expression analysis directly or those DEGs derived from the clustering analyses previously described. Result tables were downloaded from each Enrichr query database of interest if that result table contained at least one putatively brain-relevant, q-value significant enrichment being driven by at least five of the input genes (or, in cases of large gene sets, at least eight; specified in corresponding figure legends). Result tables were then collated across databases in R (see below, Code availability).

### Comparison to DEGs from prior studies

WT stage-stage comparisons for dorsal hippocampus were compared to significant DEGs previously reported between estrous stages in Table 4 of Dicarlo et al, 2017. Each direction of effect was separated into its own column (two directions by six comparisons = 12 columns). Genes listed in DiCarlo that did not meet filtering criteria for analysis in our study were removed. For the genes analyzed in both our study and in DiCarlo et al., 2017; we programmatically obtained the number of previously reported DEGs with concordant effect directions (e.g., proestrus > diestrus) in our data both without statistical thresholds and at an FDR threshold of 0.05 (see below, Code availability).

### Data availability

Data are available at GEO under accession GSE199722. Reviewer token, if needed, is yzybsiectzotpwt.

### Code availability

Code for read QC, alignment, and filtering is available from the authors on request. The raw unfiltered counts, a filtered count matrix as used for analysis, code to analyze the filtered counts in limma, and scripts for all analyses/plotting thereafter (with the exception of Enrichr analyses themselves, which were executed and collected through the Enrichr web tool) are available on Bitbucket at https://bitbucket.org/jdlabteam/workspace/projects/CHER.

## Results

### Wild-type dorsal hippocampal gene expression: sex and estrous differences

#### Gene expression between wild-type male and female bulk dorsal hippocampus does not substantially differ

As collection of this cohort of mice required some flexibility in terms of mouse age (given size and number of conditions), we first examined whether age played a substantial, statistically-adjustable role in our differential expression analyses. Using the ComBat-seq package in R, we specified the same model (see Materials and Methods) with an additional covariate of age (categorically, in months) as a potential confounding variable to be adjusted for. We compared three-dimensional PCA plots of the limma model fits with and without preceding ComBat-seq correction to determine whether age correction altered the relationships among RNA-seq samples. These plots visually suggested (Extended Data Fig. 1-1) a modest relationship between principal component 2 (PC2) and age. However, PC2 explained 12.1% of variance in the original data, while still explaining 10.3% of variance in the age-negated data, confirming that age did not substantially influence our results (i.e., only ∼2% of the cohort-wide variance was attributable to age). We therefore did not adjust for age in the analyses that follow.

**Figure 1.**
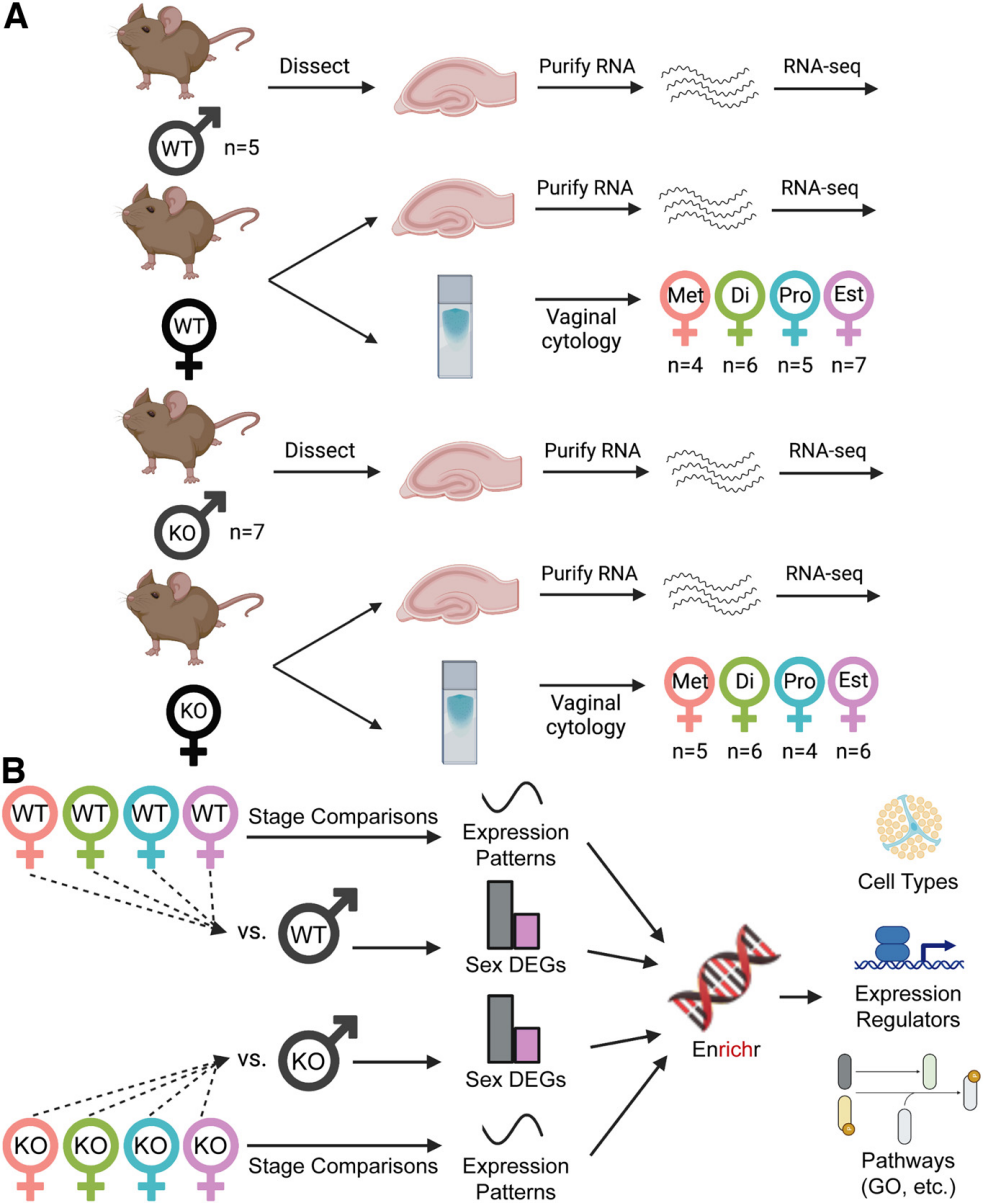
Experimental workflow. ***A***, Dorsal hippocampi from male and female adult C57/B6 wild-type (WT) and *Cnih3* knock-out (KO) mice were collected for RNA-seq (one hippocampus/mouse = one sample). Vaginal cytology was performed at time of tissue collection. Estrous stage was independently determined from cytology by two to three scorers. Final *n* for each genotype and estrous stage group are indicated in the figure. ***B***, Analytic workflow. Sequencing QC results and mouse metadata are included in Extended Data Table 1-1. Additional QC in the form of principal component analyses with and without adjustment for mouse age are in Extended Data Figure 1-1. Met = metestrus; Di = diestrus; Pro = proestrus; Est = estrus.

**Figure 2.**
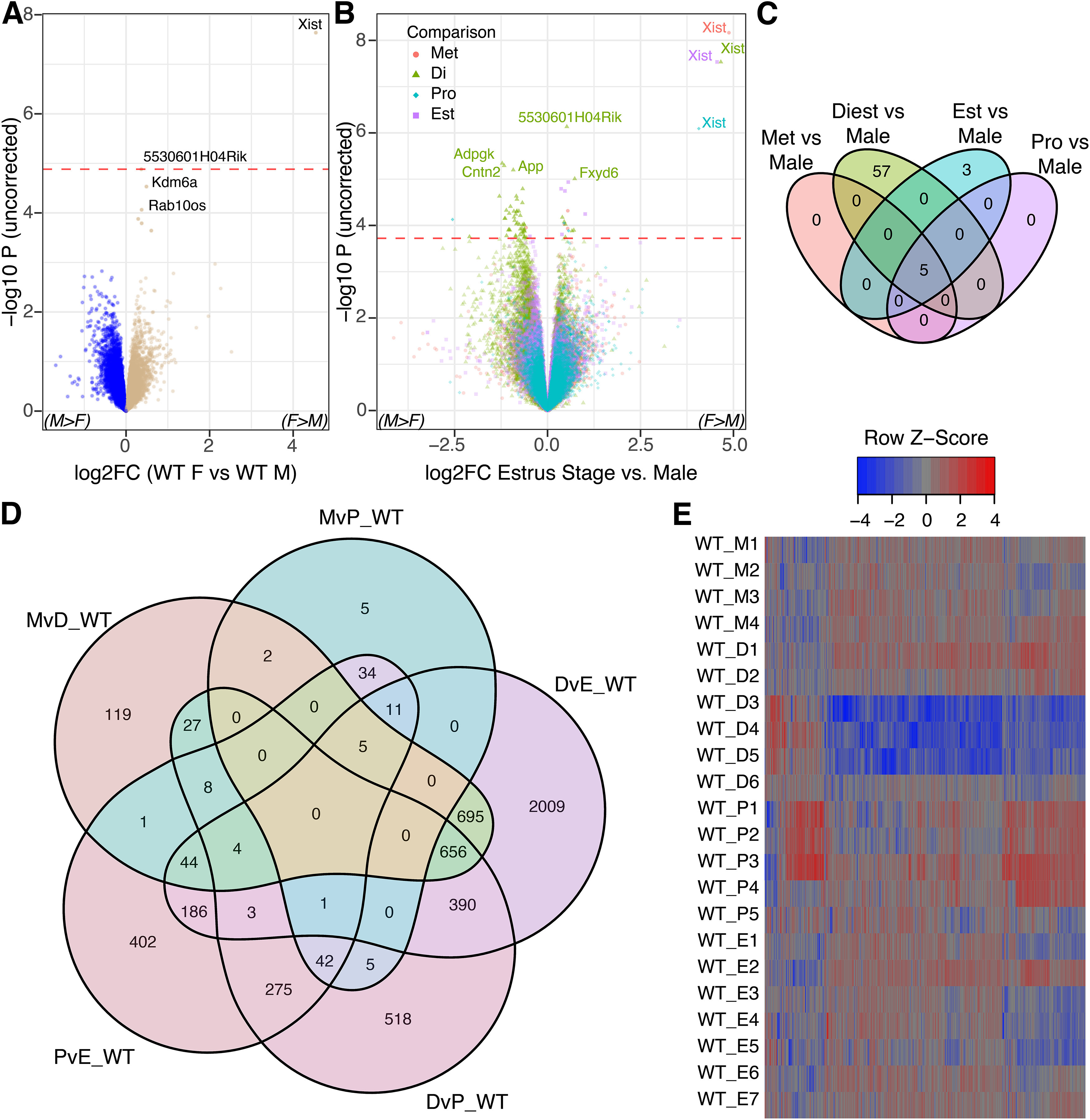
Wild-type dorsal hippocampal transcriptome varies across estrous stages. ***A***, Volcano plot illustrating the effective absence of sex-differential genes when comparing wild-type males to females combined across all stages of the estrous cycle. Female enriched genes are positive. ***B***, A joint volcano of differential expression analysis results for each estrous stage compared with males. The largest magnitude differences in gene expression are between diestrus and males. *Xist* is shown on the plot to illustrate the comparative magnitude of sex-specific gene expression from the sex chromosomes; Y-chromosome genes are excluded for scale. ***C***, Venn diagram illustrating the number of significant DEGs between males and females for each estrous stage. The majority of differential expression occurs in diestrus (see also replication of stagewise differential expression compared with a prior study of hippocampus in Extended Data Fig. 2-1). ***D***, A Venn diagram of differentially expressed genes across pairwise comparisons of estrous stages, illustrating the dependence of the female hippocampal transcriptome on estrous stage. Once again, diestrus is the most distinctive of the four stages. Full RNA-seq analysis results for comparisons in panels ***A–D*** are included in Extended Data Table 2-1. ***E***, Heatmap of samplewise expression of the same genes as in panel ***D***. Hippocampal gene expression changes across estrous stages are individually subtle but extensive in terms of the number of genes involved. Additional clustering of gene expression by genotype and sex/estrous stage is in Extended Data Figures 2-2 and 2-3. Pairwise comparisons at or near statistical significance for three genes were directionally verified and significant by qPCR using the same RNA as sequenced (Extended Data Fig. 2-4).

We first tested the 16,168 genes in our wild-type (WT) dorsal hippocampal RNA-seq data for female gene expression differences from male, examining both estrous-naive and stage-specific differences in gene expression (Figs. 1*B*, 2; Extended Data Table 2-1*A*). At the level of sex alone, we only detected six differentially expressed genes (DEGs; FDR < 0.05, no log fold-change threshold), all from the sex chromosomes (Fig. 2*A*). Examining nominally significant DEGs with a log fold-change (FC) exceeding 1.5 revealed four additional genes, including *Depp1* (female-upregulated, logFC 2.3, *p* < 4 × 10^−3^) and *Avpr1b* (female-upregulated, logFC 2.13, *p* < 2 × 10^−3^). In all, the “net” female dorsal hippocampal transcriptome did not diverge appreciably from male.

10.1523/ENEURO.0153-22.2023.f1-1 Extended Data Figure 1-1 3D principal component analysis (PCA) of highly variable gene expression data analyzed with versus without removal of age effects by the ComBat-seq package. Expression values were filtered to those with a standard deviation of ≥1 for PCA calculation and plotting. Similar perspectives shown of 3D PCA of the cohort gene expression values as (***A***) modeled by limma/voom and used throughout the paper and (***B***) as corrected for categorical age by ComBat-seq prior to modeling in limma/voom. Download Figure 1-1, TIF file.

10.1523/ENEURO.0153-22.2023.f1-2 Extended Data Figure 1-2 3D PCA of highly variable WT gene expression reveals no role of parental genotype in observed DE patterns. Expression values were filtered to those with a standard deviation of ≥1 for PCA calculation and plotting of WT samples. CNIH3: one parent was heterozygous for the *Cnih3* mutation (and offspring was mutation-negative); WT B6: both parents were vendor-purchased B6 or their in-house, inbred descendants. Download Figure 1-2, TIF file.

10.1523/ENEURO.0153-22.2023.tab1-1 Extended Data Table 1-1 RNA-seq QC results (read depth, and percent of reads mapped, uniquely mapping, and multimapped) and sample metadata including age in weeks. Download Table 1-1, XLS file.

10.1523/ENEURO.0153-22.2023.f2-1 Extended Data Figure 2-1 Replication of WT estrous stage pair DE directionality between the present study and DiCarlo (2017). Each *x*-axis value represents one direction of expression change between the first and second listed stages (e.g., P > E signifies genes upregulated in proestrus relative to estrus; P < E signifies downregulation in proestrus relative to estrus). All six comparisons (12 directional changes) were examined in WT both here and elsewhere (DiCarlo, 2017). See Materials and Methods, Comparison to DEGs from prior studies, for additional details. Red bars indicate number of genes analyzed in both studies; green indicates genes with the same directional relationship as previously published (DiCarlo, 2017) regardless of statistical significance, while blue indicates the number of genes that were also significantly DE in the present study at FDR < 0.05. M, metestrus; D, diestrus; P, proestrus; E, estrus. Download Figure 2-1, TIF file.

10.1523/ENEURO.0153-22.2023.f2-2 Extended Data Figure 2-2 Three-dimensional PCA of all analyzed samples using variably expressed genes including sex chromosomes. Download Figure 2-2, TIF file.

**Figure 3.**
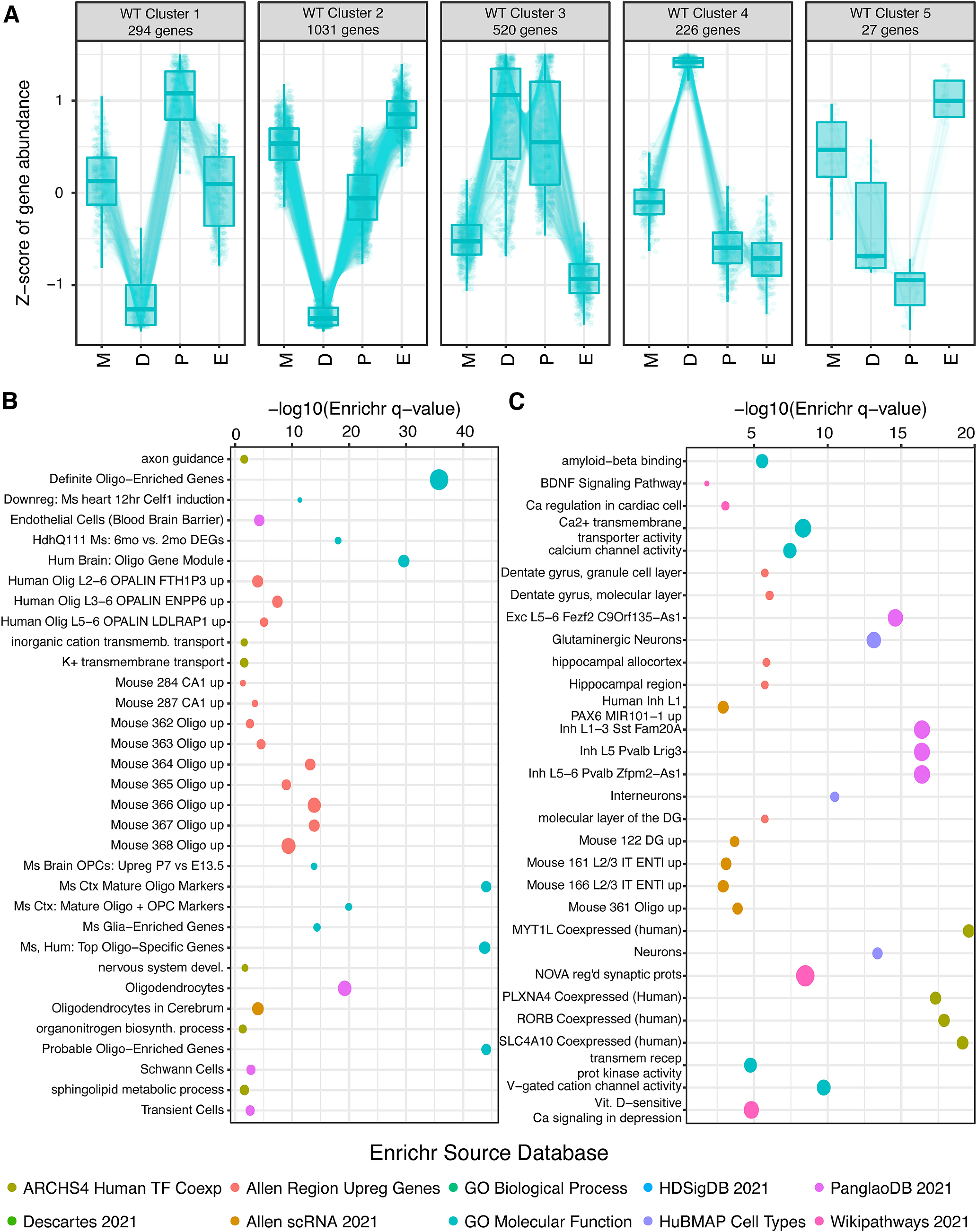
Wild-type female cyclic hippocampal gene expression patterns across estrous. ***A***, Patterning analysis of gene expression for DEGs between any pair of stages at FDR < 0.01 identifies five clusters of expression fluctuation across the estrous cycle. ***B***, Selected Enrichr analysis results for cluster 1 from panel ***A***. Specific terms are highlighted in the plot, all with log odds ratio (OR) of the cluster gene set >2.5, all enrichment (uncorrected) *p*-values < 0.05, with more than or equal to eight genes from the cluster included in the enriched annotation term. Point colors indicate which Enrichr dataset each term comes from, while point sizes are scaled to the log OR. ***C***, Selected Enrichr analysis results for cluster 2 from panel ***A***. All terms meet the same filtering criteria as in panel ***B***. Gene-to-cluster assignments and complete Enrichr results are given in Extended Data Table 3-1.

10.1523/ENEURO.0153-22.2023.f2-3 Extended Data Figure 2-3 Three-dimensional PCA of all analyzed samples using variably expressed autosomal genes. Download Figure 2-3, TIF file.

**Figure 4.**
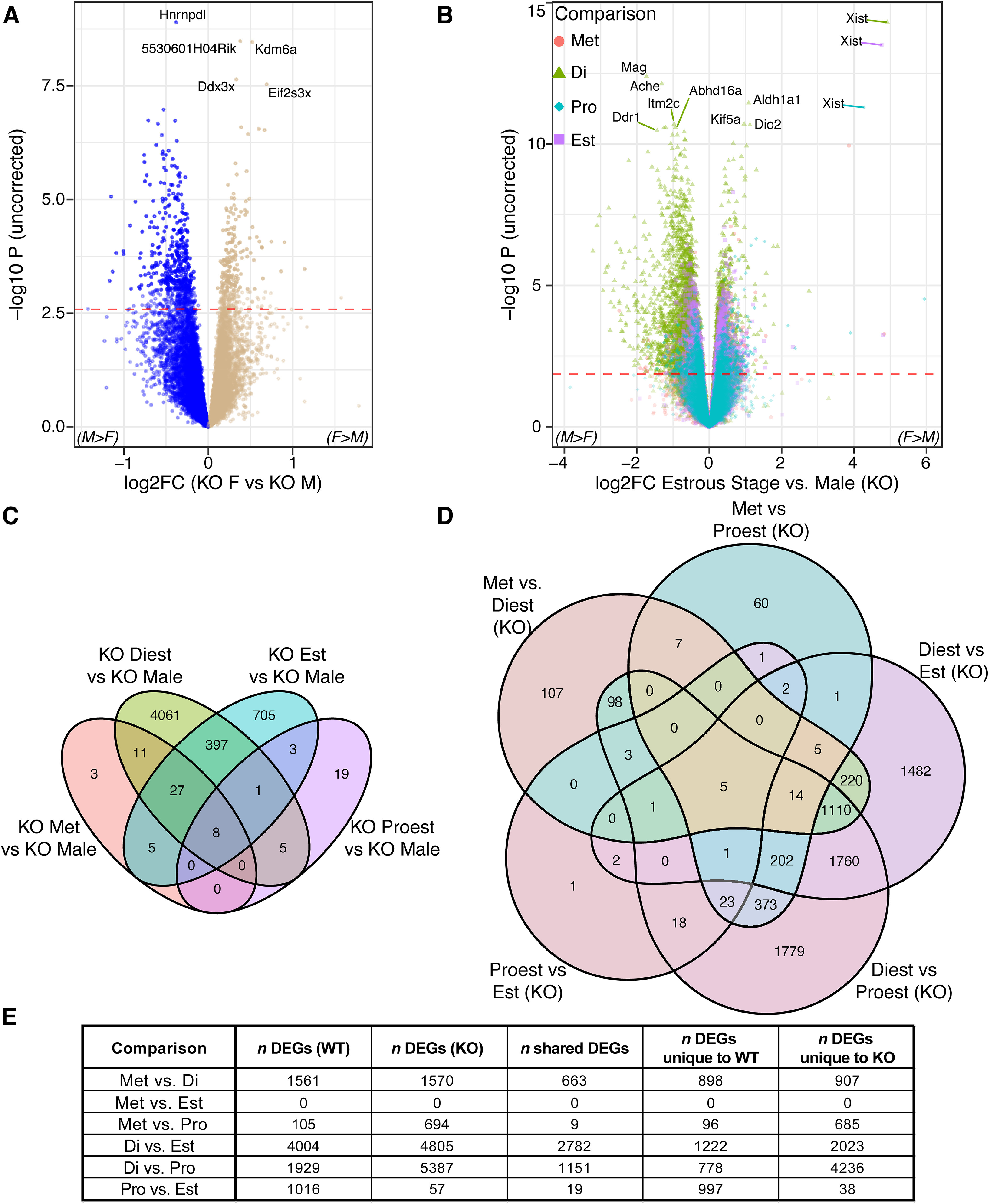
*Cnih3* knock-out hippocampal transcriptome shows greater differences between sexes and estrous stages. The knock-out effect on *Cnih3* exons shown in Extended Data Figures 4-1 and 4-2 confirmed the KO genotype for all assessed mice. ***A***, At the level of males compared with all females, the *Cnih3* knock-out line shows a substantial increase in the number of significant DEGs compared with WT (Fig. 2*A*). ***B***, A joint volcano of differential expression analysis results for each KO estrous stage compared with KO males. The largest magnitude differences in gene expression are between diestrus and males, consistent with the WT pattern of diestrus constituting the most distinct transcriptional state. ***C***, Likewise, the greatest number of DEGs between males and an estrous stage are found at diestrus, as shown in the Venn diagram of significant genes from the comparisons in panel ***B***. ***D***, Diestrus is likewise the most distinctive transcriptional state within *Cnih3* KO females, illustrated in a Venn of genes significantly DE between any two stages, where the preponderance of stage-pair-specific DEGs correspond to a diestrus comparison. ***E***, Table summarizing estrous stage-stage comparisons in WT and KO by number of total DEGs in each genotype and number of shared DEGs across the two genotypes for each stagewise comparison. Full RNA-seq analysis results for comparisons in panels ***A–D*** are included in Extended Data Table 4-1. *Limma* adjusted RNA-seq log2(CPM) values after low-expression filtering are in Extended Data Table 4-2 for the entire cohort of WT and KO mice.

10.1523/ENEURO.0153-22.2023.f2-4 Extended Data Figure 2-4 qPCR validation of three DEGs (*Otx2*, *Prlr*, *Prl*) between estrous stages. Bonferroni corrected *p*-values (correcting for twelve total comparisons across the three genes) are shown for each comparison preselected for replication by qPCR. Download Figure 2-4, TIF file.

10.1523/ENEURO.0153-22.2023.tab2-1 Extended Data Table 2-1 Wild-type differential expression contrast results. Sheets ***A–K*** are named according to the contrast performed and cover all genes analyzed as described in Materials and Methods. Highlighted rows signify genes we called significant (i.e., FDR < 0.05 with no logFC cutoff). Download Table 2-1, XLS file.

As prior experiments with *Cnih3* have illustrated, male-female differences can be influenced by the stage of the estrous cycle (H.E. Frye et al., 2021). Therefore, we performed comparisons between WT male and WT females for each estrous stage separately, identifying 65 unique genes as significant across the four comparisons (Fig. 2*B*,*C*; Extended Data Table 2-1*B*,*E*). For metestrus (Met) and proestrus (Pro), and most surprisingly, estrus (Est), we only identified sex chromosomal genes as DEGs (FDR < 0.05) when compared with male. In contrast, we identified 62 significant DEGs in diestrus (Di) compared with male, 57 of which were unique to this stage (eight female-upregulated, 49 male-upregulated; these did not include *Cnih3*, consistent with qPCR reports; H.E. Frye et al., 2021). Altogether, these findings suggest that the female dorsal hippocampus only diverges to any appreciable extent from males during diestrus.

#### Gene expression changes substantially across estrous stages within WT females

We next examined the female samples alone, comparing each estrous stage to each other stage to identify genes significantly fluctuating over the course of the estrous cycle. Strikingly, we identified over 5000 unique DEGs (FDR < 0.05, no logFC threshold) between at least one pair of stages of the 6 combinations possible (Fig. 2*D*,*E*; Extended Data Table 2-1*F–K*). There were no significant DEGs between Met and Est, while the other 5 pairwise comparisons yielded 105–4004 significant DEGs each. Quantitative PCR (qPCR) performed using the RNA from this entire cohort of animals verified large-magnitude changes observed in the sequencing, confirming the accuracy of our sequencing and analysis (Extended Data Fig. 2-4).

Altogether, these findings suggest that male dorsal hippocampal gene expression diverges neither from females overall, nor appreciably from individual estrous stages. Instead, most variability in expression is constrained to females in an estrous stage-dependent manner. Diestrus appears to correspond to the most distinctive transcriptomic state of the dorsal hippocampus in that it is the most distinct from both males and other estrous stages (in terms of DEG) at respectively modest and sprawling scales.

We noted a striking increase in number of DEGs between pairs of estrous stages observed here compared with prior work. To ensure our analysis was consistent with prior studies of estrous effects on WT hippocampal gene expression, we compared our results to available published data. A prior study of WT rat dorsal and ventral hippocampus identified 37 DEGs between proestrus and estrus, validating seven of them by qPCR (Iqbal et al., 2020). Despite species and region/subregion differences between this study and ours, we observed significant DE for three of those seven genes between proestrus and estrus. We confirmed overall patterns of estrous DE by comparing our directions of effect with those of significant DEGs from a prior study of mouse hippocampus (DiCarlo et al., 2017). We noticed relatively limited FDR < 0.05 replication between the current data and that from DiCarlo. We interpreted comparisons in terms of DE significance cautiously, as we could not determine whether the DEGs reported in that study were called with a fold-change minimum, and as the prior study’s results were generated using the DEseq package, which has been shown to be prone to false positives in large/multivariate sequencing analyses (Li et al., 2022), and does not account for genewise variance patterns, in contrast to the limma-voom analysis employed here (Liu et al., 2015). However, when solely comparing directional relationships without statistical thresholds imposed on our data for the two largest gene sets from their data (proestrus > estrus; diestrus < proestrus), 100% of our effect directions were in agreement (Extended Data Fig. 2-1). We therefore attribute the sharp increase in estrous-regulated genes detected here to our analysis strategy’s variance control, sample size, and potentially to differences in use of fold-change thresholds.

#### Gene expression patterns in WT dorsal hippocampus across the estrous cycle

Given the large number of pairwise DEGs identified between different stages of the estrous cycle, we were well-powered to cluster these genes by their rise and fall across the cycle to predict putative biological functions subject to estrous influence in the dorsal hippocampus. Characterization of these patterns resulted in a resource for hypothesis generation concerning sex differences in the dorsal hippocampus, including as a comparator for subsequent expression profiling of *Cnih3* KO. In addition to the provided results from stage-stage comparisons (Extended Data Table 2-1*F–K*), we present a series of gestalt analyses to understand these differences below.

To clump genes by their cyclic pattern of expression, we used the *DEGReport* package’s *degPatterns* function in R 4.1.2, and clustered the union of genes significant at an FDR < 0.01 (1700 genes total) from any estrous comparison above. We identified five gene expression patterns total in WT (Fig. 3*A*; Extended Data Table 3-1*A*), the majority of which fell into clusters corresponding to peak expression in diestrus (cluster 4), trough expression at diestrus (clusters 1 and 2), or peak expression at proestrus (clusters 1 and 3). We next analyzed each cluster’s genes for specific biological pathways using the *Enrichr* tool (E.Y. Chen et al., 2013). *Enrichr* annotations of note for four of these clusters (1–4) highlighted several pertinent aspects of brain and hormonal biology (Extended Data Table 3-1*B*). WT estrous cluster 1, characterized by peak expression in proestrus and trough expression in diestrus (Fig. 3*A*), was enriched for oligodendrocyte marker genes (Fig. 3*B*). (Indeed, 4/6 of the stagewise comparisons above showed differential expression of *Gal3st1*, whose protein product sulfonates carbohydrates in sphingolipids to produce sulfatide, a major component of myelin). Cluster 2, also characterized by trough expression in diestrus but with estrus-metestrus peak expression, showed enrichment for genes in “early estrogen response,” calcium signaling, glutamate receptor signaling, and axon guidance, as well as strong enrichment for inhibitory interneuron subtypes, glycinergic neurons, and all classes of glia, and finally regional enrichment for the molecular layer of the dentate gyrus (Fig. 3*A*,*C*). By contrast, WT estrous cluster 3, with peak expression across diestrus and proestrus, was enriched for protein interactors of estrogen receptor *Esr1* with only weak enrichment for dopaminergic, glycinergic, and subtype-nonspecific neuron markers (Extended Data Table 3-1*B*). Finally, cluster 4, representing genes sharply peaking in diestrus, was strongly enriched for *Sncg*+ neurons and hippocampal CA3 neurons from Allen Brain Atlas single-cell RNA-seq. Cluster four was also enriched for genes upregulated by knock-down of *RELA*, *Neurod1*, or *Mecp2*, or by overexpression of *Neurog3* (Extended Data Table 3-1*B*). As some of these TFs are known regulators of neuronal gene expression, this suggests neuronal gene expression is disproportionately altered in this cyclic manner. Reassuringly, the pathway analyses highlight CNS pathways and cell types, rather than those of other tissues, consistent with a bona fide estrous cycle impact on the brain. These findings highlight higher-order biological changes in the dorsal hippocampus occurring at different stages in the estrous cycle.

10.1523/ENEURO.0153-22.2023.tab3-1 Extended Data Table 3-1 Wild-type estrous gene cycling patterns and pattern annotations. ***A***, Pattern assignment of genes DE between any two estrous stages in WT at an FDR < 0.01. ***B***, Enrichr analysis results for each gene set defined in sheet ***A***, with all enrichments achieving at least nominal significance with an enrichment log OR > 2.5 and at least 8 DEGs overlapping those of the enriched term. Highlighted rows indicate term enrichments meeting these thresholds. Results for each gene set only are reported for Enrichr databases with nominally significant enrichment of at least one term of potential nervous system or endocrine implications. Download Table 3-1, XLS file.

### *Cnih3* knock-out effects on dorsal hippocampus

We first examined KO and WT *Cnih3* sequencing alignments to confirm that *Cnih3* exon four was indeed absent, as expected for this mouse line (Extended Data Figs. 4-1, 4-2). This mutation induces a frameshift and thus predicted loss of function in *Cnih3*. Subsequently, we examined expression of all genes for the KO mice in the same series of approaches as for WT above, which are on the whole presented in a similar structure to those above for sake of comparability. We then performed KO versus WT comparisons for each sex/estrous stage, and finally, we describe the overall key patterns of transcriptomic alterations identified in the *Cnih3* KO dorsal hippocampus.

#### Gene expression differences between *Cnih3* KO males and females are subtle but far outnumber WT sex differences

*Cnih3* KO males (*n* = 7) and females (all estrous stages considered jointly, *n* = 21) showed much starker differential expression in the dorsal hippocampus, with 849 genes detected as differentially expressed at FDR < 0.05 (Fig. 4*A*; Extended Data Table 4-1*A*). Notably, only 18.6% of these genes had an absolute logFC exceeding 0.5, suggesting the vast majority of estrous-nonspecific, sex-differential expression in the *Cnih3* KO dorsal hippocampus remains subtle in nature. By contrast, two autosomal genes showed large, FDR-significant sex effects with a logFC of > 1.5: perilipin 4 (*Plin4*) and prolactin (*Prl*), the latter having only achieved nominal significance in WT male-female DE analysis. Quantitative PCR (qPCR) performed using the RNA from this entire cohort of animals confirmed large-magnitude differential expression between several pairs of stages and between the sexes in KO mice, including for *Prl* (Extended Data Fig. 2-4).

10.1523/ENEURO.0153-22.2023.f4-1 Extended Data Figure 4-1 *Cnih3* RNA-seq read coverage in each analyzed sample. ***A***, Coverage in WT samples. ***B***, Coverage in KO samples. Download Figure 4-1, TIF file.

10.1523/ENEURO.0153-22.2023.f4-2 Extended Data Figure 4-2 *Cnih3* read coverage recapitulates the KO strain loss of exon 4 previously reported. Higher zoom of the exon 4 region of *Cnih3* is shown for (***A***) WT samples and (***C***) KO samples, with the *Cnih3* gene track and corresponding information on exon 4 shown in between (***B***). Download Figure 4-2, TIF file.

**Figure 5.**
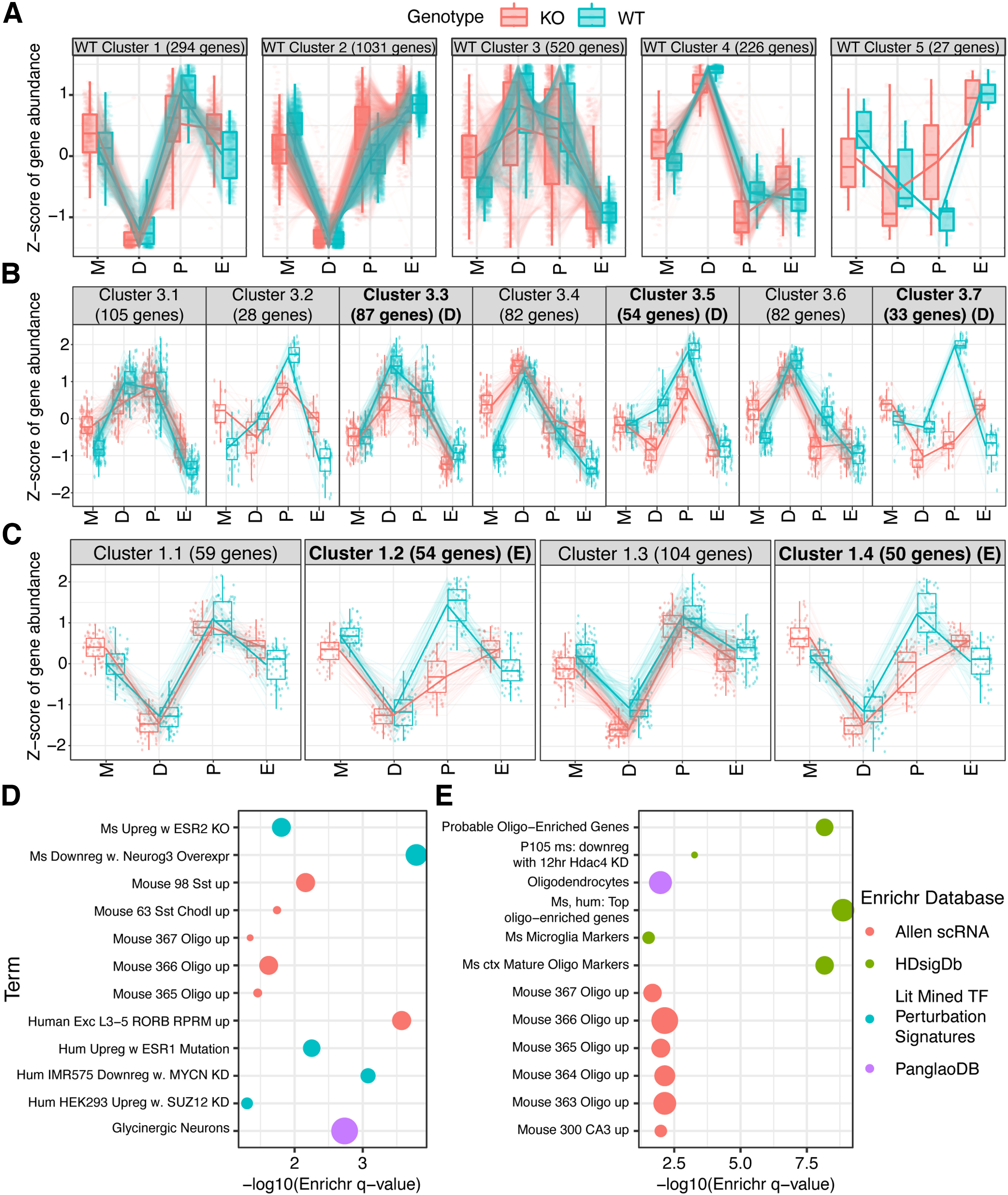
Retained and dysregulated patterns of hippocampal gene expression over the *Cnih3* KO estrous cycle. Note that daily estrous staging in a separate cohort of *Cnih3* KO animals shows unchanged cycle patterns by cytology compared with WT, with staging agreed on by two to three blinded scorers (Extended Data Fig. 5-1). ***A***, The same genes from the same five wild-type clusters of cyclic expression shown in Figure 3*A* are again shown, but now with the KO expression levels of those genes additionally plotted. ***B***, Re-clustering of WT cluster 3 using both WT and KO data identified seven subclusters (3.1, 3.2…), corresponding to a specific pattern of regulation in WT and a specific pattern in KO. Three of these clusters (3.3, 3.5, 3.7) show an attenuation of gene upregulation in KO as the cycle progress, especially in the Met -> Di and Di -> Pro transitions. ***C***, Similar re-clustering of WT cluster 1 identified four pattern subgroups, two of which also featured attenuation of upregulation at proestrus compared with WT (1.2, 1.4). ***D***, Enrichr analysis of the genes in subclusters 3.3, 3.5, and 3.7 combined highlight that they are DE in estrogen receptor perturbation experiments and enriched in oligodendrocytes and inhibitory neuron classes. ***E***, Enrichr analysis of genes in subclusters 1.2 and 1.4 combined were highly enriched for oligodendrocyte genes across several annotation sets, and suggest *Hdac4* as an upstream regulator. Filters for both Enrichr plots are the same as for Figure 3, except with a minimum term-gene set overlap of five rather than eight to account for the smaller gene set sizes of these subclusters. Gene-to-cluster assignments and unabridged Enrichr results for this analysis are in Extended Data Table 5-1. Two example oligodendrocyte genes underlying enrichment results for KO expression pattern subsets showing attenuated upregulation from diestrus to proestrus are shown in Extended Data Figure 5-2.

10.1523/ENEURO.0153-22.2023.tab4-1 Extended Data Table 4-1 *Cnih3* KO mouse differential expression contrast results, number of DEGs and degree of DEG overlap between KO and corresponding WT contrasts, and re-clustering assignments of estrous cycling genes from WT clusters 1 and 3. ***A–K***, Sheets are named according to the contrast performed and cover all genes analyzed as described in Materials and Methods. Highlighted rows indicate genes we considered DE (FDR < 0.05). ***L***, Table summarizing estrous stage-stage comparisons in WT and KO by number of total DEGs in each genotype and number of shared DEGs across the two genotypes for each stagewise comparison. ***M***, Gene clustering reassignment of those genes from WT clusters 1 and 3 when also including KO data as a second set of data points. *Orig.WT.Clust* indicates the parent cluster (i.e., from Extended Data Table 3-1*A*), while *joint.WT.KO.subcluster* indicates the subcluster that resulted (i.e., as shown in Fig. 5*C*,*D*). These are the same indexing values used to describe subclusters in the main text/figures (e.g., subcluster 1.3 has column values of Orig.Wt.Clust as 1, joint.WT.KO.subcluster as 3). ***N***, Enrichr results for the union of genes in subclusters 1.2 and 1.4 or in the union of subclusters 3.3, 3.5, and 3.7. Results for each gene set only are reported for Enrichr databases with nominally significant enrichment of at least one term of potential nervous system or endocrine implications driven by more than or equal to five DEGs and with an enrichment log OR > 2.5. Enrichments meeting these criteria are highlighted in the table. Download Table 4-1, XLS file.

10.1523/ENEURO.0153-22.2023.tab4-2 Extended Data Table 4-2 Samplewise expression data for filter-passing genes. Moderated log2 counts per million (CPM) values for all samples analyzed in the study. These values are the values used for generation of plots using expression/Z-scored expression (e.g., expression clusters). Download Table 4-2, XLS file.

Considering the estrous stage-specific sex differences in behavior and dorsal hippocampal architecture previously described in *Cnih3* KO mice (H.E. Frye et al., 2021), we also examined males compared with each stage of the estrous cycle independently (Fig. 4*B*; Extended Data Table 4-1*B–E*). Here, we also detected a far greater number of DEGs compared with the same approach in WT: an 80-fold greater number of unique DEGs across the four KO comparisons, totaling 5245, compared with the 65 from the four contrasts in WT. The overall distribution of DE events was similar to WT, albeit on a much larger scale, with 54, 4510, 36, and 1146 DEGs for Met, Di, Pro, and Est, respectively, compared with males (Fig. 4*C*,*D*). Likewise, the number of high-magnitude (log FC > 1.5) DE events for Di versus males totaled 145 in KO, versus six in WT. Altogether, these findings suggest that *Cnih3* knock-out accentuates sex-differential gene expression in both a global and estrous stage-specific manner.

#### *Cnih3* KO females retain WT estrous expression patterns outside of small sets of proestrus-stimulated genes

As in our WT data, we then performed pairwise comparisons of estrous stages in the *Cnih3* KO samples (Extended Data Table 4-1*F–K*). On the whole, these differential expression sets were comparable in size, with the exception of substantial increases in the number of FDR significant DEGs between Di-Pro and Di-Est for KO relative to WT (increases of ∼3400 and ∼800 genes, respectively; Fig. 4*E*). Despite the similar gene set *sizes,* the DEGs between the WT and KO comparisons were only ∼40–50% shared (Fig. 4*E*), additionally suggesting perturbations to ordinary estrous cycle gene expression.

To clarify whether the broad-scale cyclic patterns of gene expression across the estrous cycle we identified in WT above were intact in *Cnih3* KO mice, we Z-scored the KO expression levels of the same genes and overlaid them into the WT clustering space to compare their cyclic expression to WT (Fig. 5*A*). While many clustering patterns seemed to be generally retained, if sometimes with larger gene-set level variance in KO, there were notable discrepancies between WT and KO cycling patterns for clusters 1 and 3, two clusters defined by genes with peak expression in proestrus.

10.1523/ENEURO.0153-22.2023.f5-1 Extended Data Figure 5-1 Daily estrous cycle cytology in an independent cohort of adult *Cnih3* KO and WT females. *N* = 27/genotype. A total of 12 mice were observed over a 14-d period not including weekends, while 42 mice were observed over 11 consecutive days. Y values between stages correspond to cytologic indicators of stage transitions (e.g., diestrus-proestrus). All plotted points represent staging agreed upon by two to three independent, blinded scorers. Download Figure 5-1, TIF file.

10.1523/ENEURO.0153-22.2023.f5-2 Extended Data Figure 5-2 Example oligodendrocyte-enriched genes found in estrous pattern subclusters showing upregulation from diestrus to proestrus in WT and attenuated proestrus upregulation in KO. ***A***, *Srd5a3*. ***B***, *Gpr17*. Di = diestrus; Pro = proestrus. Download Figure 5-2, TIF file.

10.1523/ENEURO.0153-22.2023.tab5-1 Extended Data Table 5-1 Genotype differential expression contrast results. ***A***, ***C–G***, Sheets are named according to the contrast performed and cover all genes analyzed as described in Materials and Methods. Highlighted rows indicate genes we considered DE (FDR < 0.05). ***B***, Nominally significant Enrichr results for genes DE between all KOs and all WTs considered together. Highlighted rows met our full criteria for enrichment for this analysis: log OR > 2, more than or equal to eight DEGs in the listed Enrichr gene set, and nominal enrichment *p*-value < 0.05. Download Table 5-1, XLS file.

To further dissect the alterations to clusters 1 and 3, we re-performed clustering on the genes defining the WT cluster using KO and WT data combined, resulting in 4 and 7 subclusters of expression, respectively (i.e., subclusters with a specific pattern in WT and a specific pattern in KO; we call these, e.g., cluster 3.1, 3.2…). This co-visualization, and the fact that unitary WT clusters divide up into multiple parts when KO data are also considered, confirmed that the KO estrous cycle patterns of certain subsets of genes were highly divergent from their WT counterparts. In the case of WT cluster 3, our combined-genotypes analysis revealed three subclusters (3.3, 3.5, and 3.7) with attenuated gene upregulation at proestrus in KO mice (Fig. 5*B*).

To understand functional correlates of these subclusters’ combined genes, we performed Enrichr analysis, revealing oligodendrocytes and, interestingly, genes found to be upregulated in estrogen receptor knock-out datasets (Fig. 5*C*; Extended Data Table 4-1*M*,*N*). Likewise, we noticed an attenuation of gene upregulation in KO mice over the diestrus-estrus stages for subclusters 1.2 and 1.4 (Fig. 5*D*), whose parent cluster had shown enrichment for oligodendrocyte marker genes and potassium channels. Enrichr analysis of these two combined subclusters strikingly revealed that 52 of these 104 genes were in “Mouse Cortex Mature Oligodendrocyte And Progenitor Cell Markers” as defined previously (Doyle et al., 2008); the 104-gene set was also enriched in an additional oligodendrocyte marker list similarly mined from literature (Cahoy et al., 2008), and in various combinations of oligodendrocyte genes identified by single cell RNA-seq (Fig. 5*E*; Extended Data Table 4-1*M*,*N*). Among these genes were *Gpr17,* a factor for oligodendrocyte precursor maturation and myelination (Y. Chen et al., 2009; Lu et al., 2018), and *Srd5a3*, a 5-α reductase that converts testosterone to dihydrotestosterone (DHT; Uemura et al., 2008; Extended Data Fig. 5-2). These findings very strongly suggest that the *Cnih3* KO mouse has specific deficits in oligodendrocyte gene upregulation in response to proestrus.

### WT-KO differential expression is unremarkable within sex/estrous stage, against expectations

**Figure 6.**
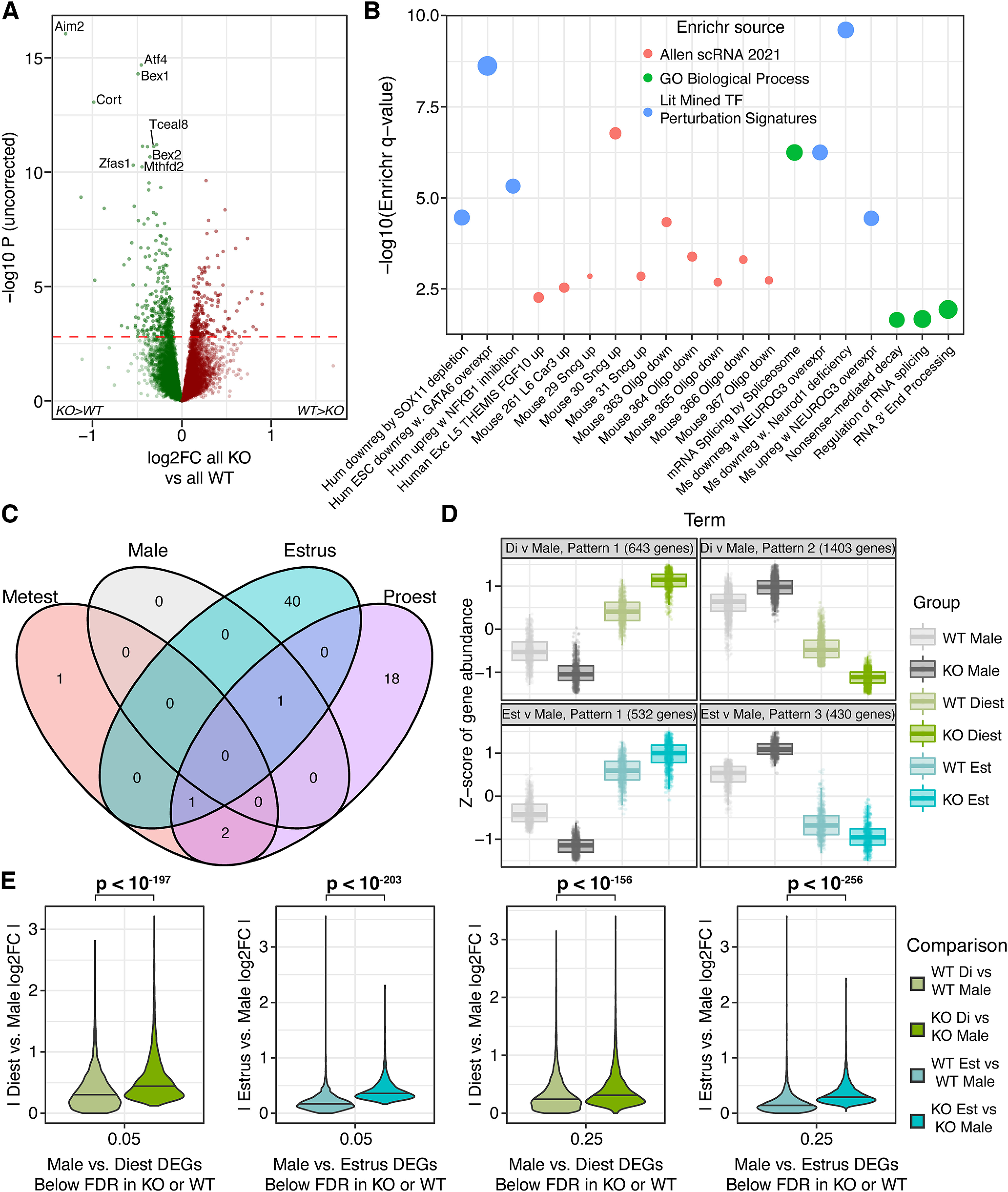
Global features of *Cnih3* KO: Splicing, synuclein, SST, subsurface neurons, and sex difference accentuations. ***A***, Comparing all KOs to all WTs, regardless of sex or estrous stage, identifies hundreds of significant DEGs. ***B***, Global DEGs in *Cnih3* KO are enriched for multiple neural subtypes of the mouse brain marked by synuclein γ (*Sncg*), deep layer excitatory neurons, *Sst*-expressing interneurons, and ontology terms related to several forms of RNA processing. Notably, the same oligodendrocyte subtypes identified as *enriched* for genes dysregulated in the KO estrous cycle are depleted (i.e., lowly-expressed) in the global DEGs of the KO mouse. All terms plotted were nominally significant for enrichment and met filters for a minimum log OR of two and a minimum of eight DEGs overlapping with the annotation term set. ***C***, A surprisingly small number of DEGs are identified by comparing single estrous stages or males between KO and WT. (Stages not shown have 0 significant DEGs.) ***D***, Analysis of genes DE between KO males and a KO estrous stage at FDR < 0.01 showed several sizable groups of genes where *Cnih3* KO accentuated a normal sex difference seen in WTs–that is, a greater magnitude of effect in KOs while with the same effect direction as found in WT. The illustrated gene sets show a larger difference between KO males and KO females (in opaque colors) compared with the same male-stage pairs in WT (translucent colors) at the illustrated stages. ***E***, The range of absolute log2 fold-changes for the union of DEGs from WT and KO male-diestrus comparisons or from WT and KO male-estrus comparisons at multiple FDR cutoffs. At all FDR thresholds examined, the absolute magnitude of DE is significantly (Wilcoxon test) greater in the KO comparison, indicating virtually global potentiation of estrous stage-specific sex-differential expression in KOs. The RNA-seq analysis results of global KO-WT comparison, gene-to-cluster assignments, and unabridged Enrichr results for male-versus-estrous stage effect patterns in WT versus KO are in Extended Data Table 6-1.

While above we indirectly compared each contrast in KO to the same contrast in WT, we also directly contrasted the WT and KO mice to better understand effects of the knock-out on dorsal hippocampal gene expression. Contrasting all KO and WT samples, regardless of sex, identified 514 significant DEGs (35 with an absolute logFC > 0.5; Fig. 6*A*), again suggesting overall subtle effects of the *Cnih3* KO on gene expression (Extended Data Table 5-1*A*). *Enrichr* analysis of these 514 genes revealed highly significant overlap with genes from dozens of brain-relevant transcription factor perturbation experiments, including *Neurod1* knock-down and human cell culture *GATA6* overexpression. This gene set was also highly enriched for protein interactors of estrogen receptor α (*Esr1*). *Cnih3* KO DEGs were also enriched for neuronal markers including those of *Scng-*expressing interneurons. Intriguingly, and in direct contrast to oligodendrocyte genes with dysregulated cyclic expression patterns in KO, WT versus KO DEGs were instead enriched for genes not expressed in oligodendrocytes relative to other Allen Atlas single-cell RNA-seq cell types (Fig. 6*B*; Extended Data Table 5-1*B*).

10.1523/ENEURO.0153-22.2023.tab6-1 Extended Data Table 6-1 Patterns of altered sex-differential expression between WT and *Cnih3* KOs for diestrus and estrus. ***A***, Number of genes significantly (FDR < 0.05) DE in both genotypes for male versus stage, stage versus stage, and male versus all female comparisons. ***B***, Cluster assignment of KO male versus KO diestrus DEGs for expression patterns across WT male, KO male, WT diestrus, and KO diestrus. Two such patterns are shown in Figure 6*D*. ***C***, Cluster assignment of KO male versus KO estrus DEGs for expression patterns across WT male, KO male, WT diestrus, and KO diestrus. Two such patterns are shown in Figure 6*D*. ***D***, Combined Enrichr annotations for diestrus-male KO DEGs with KO/WT sex DE patterns 1 or 3 as listed in sheet 6A and for estrus-male KO DEGs with KO/WT sex DE patterns 1 or 3 as listed in sheet 6B. Also listed are enrichments found for the union of DEG sets from a male-diestrus and a male-estrus comparison where the genotypes’ DE pattern was similar for the two stages (labels reading “Union of…”). Highlighted rows met our described criteria for enrichment in this particular analysis (≥8 DEGs overlapping with Enrichr term, Enrichr OR ≥ 2, nominal *p*-value < 0.05). ***E***, Listing of each term enriched in at least one of the six cross-genotype estrous stage-versus-male DEG pattern sets above, listing which pattern(s) were enriched for a given term. Download Table 6-1, XLS file.

Subsequently, we performed within-sex/estrous stage comparisons of the two genotypes to better elucidate the effects of *Cnih3* knock-out, given the estrous stage-specific behavioral differences previously seen in these mice (H.E. Frye et al., 2021). Given the extremely large increase in the number of sex- and male-estrous DEGs within KOs, we expected to see comparable numbers of genotype-differential genes within each group. Shockingly, however, only 1–42 FDR-significant DEGs were identified in the genotype comparisons (Extended Data Table 5-1*C–G*), totaling 63 unique genes (Fig. 6*C*), with the most differences seen between WT and KO at proestrus. Surprisingly, we observed only one DEG between WT and KO diestrus, despite this stage being responsible for the most DEGs between male and other estrous stages in both WT and KO mice.

#### *Cnih3* KO results in accentuated sex-differential expression compared with WT, especially in diestrus

The findings thus far are surprising when considered in conjunction. Specifically, how is it that KO male versus estrous stage comparisons yielded far more DEGs than did the respective WT comparisons (Extended Data Table 4-1*L*), while comparing single estrous stages across genotypes yielded a paucity of DEGs (Fig. 6*C*)? We hypothesized that our findings could be because of subtle KO effects with opposite directions of effect in males and females at certain estrous cycle stages; in other words, we hypothesized that statistically small shifts of KO male expression relative to WT male expression concurrent with statistically small shifts of KO estrous stage expression relative to the same stage in WT could result in more potent DE events between KO males and a given KO stage. Figure 6*D* confirms this to be the case.

To examine whether this hypothesis was valid at the level of KOs, we first examined the consistency of DE between the two genotypes for each male-stage and stage-stage comparison, as well as the male-all-females comparison. Across these 11 contrasts, 4708 of the 4709 DE events achieving significance (FDR < 0.05) in both genotypes had consistent directionality (Extended Data Table 6-1*A*), demonstrating the internal reproducibility of the most robust DE patterns, and confirming that *Cnih3* KO did not reverse sex/estrous effects on gene expression. We again used the degPattern algorithm to visually compare WT expression of KO male-estrous DEGs. Consistent with our hypothesis, we see that KO DEGs generally show exaggerated effect magnitudes relative to their WT peers. The overall conclusion is that KOs have accentuated sex differences across large portions of the dorsal hippocampal transcriptome (Fig. 6*D*; shown are the two KO estrous stage-male comparisons with the most DEGs; Extended Data Table 6-1*B*,*C*). Enrichr analyses of genes following each pattern for these two male-stage comparisons (such as male>diestrus in WT with male upregulation and female downregulation in KO) are provided in Extended Data Table 6-1*D*, and recurring terms across genes with different patterns are tabulated in Extended Data Table 6-1*E*.

To confirm accentuated sex differences in the KOs, we also examined all genes differentially expressed in either genotype between males and diestrus or between males and estrus, and plotted their absolute log2 fold change values for both WT and KO mice (Fig. 6*E*). At any FDR thresholds tested, a nonparametric Wilcoxon test identified an extremely significant increase in the magnitude of the absolute sex differences in the KO compared with those seen in WT. This confirms the observation of a general net increase in sex-differential expression between *Cnih3* KO males and KO females during diestrus and estrus.

## Discussion

We had a variety of motivations for specifically conducting a study on estrous-regulated gene expression in the dorsal hippocampus of both WT and *Cnih3* KO mice. Prior work has demonstrated the influence of estrous stage on both hippocampal physiology and spatial learning (C.A. Frye, 1995; Warren and Juraska, 1997), highlighting the important influences of reproductive physiology on behavior and hippocampal activity. The dorsal hippocampus is an important site of integration of sex hormone and stress signaling (Bao et al., 2006; Frick et al., 2015; Padilla-Coreano et al., 2016); stress plays key roles in drug reinstatement (McKee et al., 2015) and likely psychotic disorders (Walker et al., 2013), and interestingly, is also influenced from upstream by sex through the HPA axis (Lund et al., 2004). The dorsal hippocampus is therefore of special interest in *Cnih3* mutant mice because of the association of *CNIH3* to addiction phenotypes and schizophrenia in humans (Drummond et al., 2012; Nelson et al., 2016) and reported mouse knock-out interactions with sex and estrous affecting hippocampal learning phenotypes and glutamatergic signaling pathways (H.E. Frye et al., 2021). We thus conducted a well-powered study to understand the transcriptional effects of estrous on dorsal hippocampal gene expression in both wild-type and *Cnih3* KO animals. Our results provide valuable insights to help future studies to dissect the interactions and mechanisms for sex/hormone-dependent *Cnih3* roles in behavioral learning and substance use disorder risk.

We found that on average, the brain is well buffered against sex differences in expression, with the WT gene expression showing few differences between males and females (mostly sex chromosomal). Surprisingly, we only detect DE for a handful of genes known to escape X-inactivation in mouse brain (Berletch et al., 2015) or previously reported to be sex-differentially expressed in mouse hippocampus (Vied et al., 2016; Bundy et al., 2017). This discrepancy may be because of the low likelihood of the prior estrous-agnostic sex DE studies having a balanced representation of estrous stages because of the brevity (hours) of certain stages, like estrus, and extended duration of other stages, like diestrus. Supporting this notion, a recent examination of four prior mouse hippocampal sex DE studies (Ocañas et al., 2022) identified only eight consensus sex chromosomal DEGs, all of which are in the top 11 DEGs from our WT male-female comparison as ranked by FDR.

Meanwhile, at specific stages of the estrous cycle, females differed more substantially from males. Likewise, estrous stages also showed differential expression between one another; in comparing single stages against one another or to males, diestrus was consistently the most transcriptionally distinct state from males and from other stages of the estrous cycle. Consistent with our findings of a distinctive diestrus hippocampal transcriptome, and perhaps explaining discrepancies with prior sex DE studies, it has recently been shown that chrX chromatin structure in mouse ventral hippocampus is similar between proestrus females and males, but substantially different from both at diestrus (Rocks et al., 2022).

We examined data from wild-type females and identified various changes across estrous cycle phases that may have interesting biological relevance to estrous cycle specific changes in dorsal hippocampal physiology and behavior. For genes in cluster 1, with trough expression in diestrus and peak expression in proestrus–a period over which estradiol goes from lowest to highest, and progesterone starts high and begins to decrease, we observed enrichment in oligodendrocyte markers. Myelin, which is made by oligodendrocytes and increases efficiency of synaptic transmission, has been shown to increase after increased neuronal activity in the motor cortex (Gibson et al., 2014), and this was necessary for the motor function enhancement seen in their system. It is interesting to note that long-term potentiation (LTP), which is characterized by increased neuronal transmission, is enhanced during proestrus (Warren et al., 1995) with some reporting improved object-based spatial learning during this phase (C.A. Frye, 1995). Thus, it is possible that changes in myelination might help support this increased LTP and learning. For genes in cluster 2, with peak expression in estrus/metestrus and trough expression in diestrus, a period over which estradiol declines and fluctuates from its initial peak at estrus, while progesterone starts low and increases, ,we observed enrichment in genes involved in calcium and glutamate receptor signaling. Previous research has shown that estradiol improves recognition and spatial learning, and increases hippocampal spine density in CA1 (Woolley and McEwen, 1993), which requires calcium and glutamate receptor signaling. Thus, it will be interesting to investigate whether the receptors upregulated here mediate improved spatial learning seen at this phase. Regardless, our results suggest that behaviorists may wish to measure estrus state and include it as a covariate in their analyses, especially when assessing behaviors related to the dorsal hippocampus.

We then examined data from *Cnih3* knock-outs in the same manner, identifying a much more marked extent of sex-differential expression when clumping all estrous stages together, and likewise between males and single stages. Using our WT estrous cycle gene expression patterns, and given the estrous-stage-specific behavioral changes previously observed in *Cnih3* KO mice (H.E. Frye et al., 2021), we examined whether KO expression patterns deviated from WT over the estrous cycle. We indeed identified specific subsets of genes with blunted upregulation in KO over estrogenic stages of the cycle, especially proestrus. These dysregulated gene subsets overlapped with markers of oligodendrocytes, glycinergic neurons, and somatostatin (SST) interneurons, indicating these cell types might be most impacted by *Cnih3* mutation. Pathway analysis indicated these genes were disproportionately downstream of a handful of regulators including *Hdac4*, *Klf4*, *Neurog3*, and the estrogen receptor *Esr1*, suggesting the consequences of *Cnih3* mutation might work through these molecular pathways. Consistent with hippocampal biology and its role in memory, *Hdac4* and *Neurog3* are involved in regulating dendritic morphology (Simon-Areces et al., 2010; Litke et al., 2018), while *Klf4* plays a role in regulating neural stem cell self-renewal (Qin et al., 2011). However, more work would need to be done to test specific roles of these genes downstream of *Cnih3* KO.

*Cnih3* was of interest because human genetic association studies suggest polymorphism in this region may be protective against opioid dependence (Nelson et al., 2016), a disease that involves hijacking of normal reward mechanisms including learning and memory (i.e., dorsal hippocampal) processes. CNIH3 has been shown to bind AMPARs and alter synaptic AMPAR trafficking, gating, and signaling (Schwenk et al., 2009; Coombs et al., 2012; Shanks et al., 2014; Brown et al., 2018). An initial study of hippocampal slice physiology phenotypes suggested little function for *Cnih3*, except in the context of co-deletion of *Cnih2*: when both proteins were deleted, physiological studies in the acute slices revealed a phenocopy of several aspects of GluA1 KO (a subunit of AMPARs), including altered mEPSC amplitude and kinetics, and deficits in long-term potentiation (Herring et al., 2013). However, behavioral effects were not assessed; notably, these slices were all generated from prepubescent animals, as is standard in the field, precluding characterization of adult *Cnih3* function and hormonal influences on it. Finally, a recent investigation of hippocampal learning and memory function via Barnes maze in *Cnih3* KO mice revealed no main effects of genotype initially, but a surprising amount of variance in females. Subsequent exploration of this led to the discovery of estrous-stage specific effects of both *Cnih3* KO and hippocampus-specific *Cnih3* overexpression (H.E. Frye et al., 2021). Furthermore, synaptic physiology (in adult slices) as well as biochemical and immunofluorescent analyses of synapses revealed stage specific alterations in dorsal hippocampal properties across multiple levels. All of these results led us to the hypothesis that *Cnih3* in some way buffers against hormone-dependent sex differences, with the loss of the protein unmasking deficits in KO females.

We therefore directly examined expression differences in WT versus KO altogether and between males or estrous stages. The 514 genes we identified as differentially expressed between genotypes highlighted some shared enrichments with estrous-(dys)regulated gene sets at the level of cell types (SST interneurons, *Scng*-expressing neurons) and transcriptional regulators (*Neurog3*); however, most functional enrichments were distinct, spanning several forms of RNA processing and transport, and candidate upstream regulators. These included *Neurod1*, *Hsp90*, and, interestingly, X-binding protein 1 (*Xbp1*). When we compared single estrous stages or males across genotypes, however, we identified very little differential expression. The combined observation of broader differential expression between male and estrous stages within KOs compared with within WTs, despite the absence of genotype differences, suggested to us that subtle changes were being induced by *Cnih3* KO to accentuate sex-differential expression. Indeed, we observed that the magnitude of sex-differential expression at diestrus or estrus was consistently greater in the KO (Fig. 6*E*), confirming our hypothesis that *Cnih3* buffers against excess gene-regulatory responses to cycling sex hormones.

Our study did have several limitations. Some previous studies examining estrous stage and hippocampal biology have performed extensive estrous staging across multiple cycles (Woolley and McEwen, 1992; Jaric et al., 2019), whereas here, we only performed cytologic staging on two consecutive days (the day before tissue collection and at tissue collection), which could potentially lead to inclusion of brains from mice with irregular cycling. Additionally, the cytologic diestrus phase consists of endocrinologically distinct early (low estrogen) and late (rising estrogen) phases. Future studies using the *Cnih3* global KO mouse line would benefit from serum hormonal profiling of estrous cycling to examine potential for gonadal-endocrine mechanism of the KO effect. Nonetheless, observations from 11 to 14 d of daily estrous staging in independent WT and KO mice (*n* = 27 per genotype) suggest that, cytologically, cycling is intact in KO animals (Extended Data Fig. 5-1).

Our sample clustering (Fig. 2*E*) suggests that a portion of our WT diestrus mice may have been split between two transcriptional states, which most likely represented these endocrine subphases. We note, however, that the two subclusters of WT diestrus samples are similarly sized, such that our overall data for WT at diestrus should represent the composite cytologic stage. Additionally, three-dimensional principal component analysis plotting of genes with highly variable expression across groups (≥1 SD) showed that this pattern was not unique to diestrus when either including (Extended Data Fig. 2-2) or excluding (Extended Data Fig. 2-3) sex chromosomes. Finally, we were deliberately permissive in our thresholds for calling DEGs (only requiring an FDR < 0.05 without an additional fold-change cutoff). While this procedure will result in detection of some smaller magnitude changes at the level of single genes, it aids our gestalt analysis by casting a broader net for genes subject to any degree of significant fluctuation across the estrous cycle.

Altogether, we deeply characterize dorsal hippocampal gene expression patterns across the estrous cycle in WT mice, characterize the *Cnih3* KO dorsal hippocampal transcriptome, and identify a surprising potentiation of sex-differential gene expression in this knock-out line. The data and supplements from these analyses provide extensive gene annotations for WT regulatory patterns, their dysregulation in *Cnih3* KO, and a well-powered dataset illustrating the role of estrous stage in defining sex-differential gene expression. Thus, these analyses and data provide a resource for the study of sex- and estrous-differential gene expression in the mouse dorsal hippocampus.

## Synthesis

Reviewing Editor: Erica Glasper, The Ohio State University Decisions are customarily a result of the Reviewing Editor and the peer reviewers coming together and discussing their recommendations until a consensus is reached. When revisions are invited, a fact-based synthesis statement explaining their decision and outlining what is needed to prepare a revision will be listed below. The following reviewer(s) agreed to reveal their identity: Cynthia Vied, Xin-Ming Ma.

<b>Manuscript Synthesis</b>:

While the reviewers still agree that the work is important, this revision still fails to address the major concern that very few differentially expressed genes (DEGs) were observed between sexes when previous studies have published data reporting folds more. More so, reviewers still have concerns related to the experimental methods and/or approaches that were previously pointed out. The authors are encouraged to address these remaining concerns in a revised manuscript. If possible, please submit two versions of the revised manuscript (1 with tracked changes and 1 without tracked changes). The significant amounts of line editing, while appreciated, hindered some readability.

*When appropriate, I have quoted individual reviewers - these are indicated by italicized text.

Major Concerns:

1. Please provide additional information regarding the *Cnih3* KO mouse line. Information should include the length of time this line has been separate from the B6 line as well as whether the line is maintained by backcrossing to B6.

2. The small sample size is a concern, especially given the large age range between mice. Were sufficient power analyses performed to suggest that the N size was appropriate for this study.

3. While the authors discuss the 2-day protocol for determine estrus stage, this was not appropriate. Likewise, peripheral estradiol concentrations were not assessed, which would have been helpful to validate the estrus stages. This is a limitation of the study and should be considered as such in the Discussion of the revised manuscript. This, along with point #2 above, may significantly alter results.

4. Please report whether deleting *Cnih3* disrupted the estrus cycle.

5. Please explain why the age range of the mice, from 76 to 166 days old, was so different at the time of sacrifice. Be mindful that differential expression of B6 mice between 1, 2, and 4 months of age has been demonstrated by Bundy, et al., 2017.

6. A remaining concern is the report of very few (n=6) DEGs observed between WT male and female tissues even though other groups have reported many more than this in the hippocampus of B6 mice. The existing literature should be sufficiently discussed. For example, “there are X chromosome genes that have been seen by other groups as DEGs and are known to escape X-inactivation that are not DE in this WT male vs female comparison.”

7. Discussion of the DiCarlo et al study throughout the text (and within the response to the reviewers):

a. The authors description of the DiCarlo et al methods are inaccurate. DiCarlo et al used an FDR-adjusted p-value of <0.05. Please refer to the article, paying particular attention to the following text from the methods: “A count table was made for all 47 samples. DESeq (RRID:OMICS_01306; https://bioconductor.org/packages/devel/bioc/ html/DESeq.html; Anders & Huber, 2010) was used to determine statistically significant differentially expressed genes (at a False Discovery Rate (FDR) adjusted p of <0.05, using the Benjamini-Hochberg adjustment) for pairwise comparisons (proestrus to estrus, estrus to metestrus, metestrus to diestrus, diestrus to proestrus, proestrus to metestrus, estrus to diestrus) within each brain region.”

b. The authors state that the present study and the DiCarlo study agree or that the data were replicated. These statements should be checked for accuracy.

c. ‘Although these two studies have a similar design in the estrus stages, and the same genetic background in mice, there is a discrepancy between the findings in DEGs between these two studies. DiCario identified 46 DEGs at Proestrus vs Diestrus in the hippocampus (Table 4). However, among the DEGs identified at the same stages Proestrus vs Diestrus in the current study, only 2 DEGs in Table 2j-WT Diest vs WT Pro of current study are found in the list of DiCario’s 46 DEGs, 10 of 46 DEGs were not found, and 34 of 46 DEGs in the DiCario’s list are not significant with P> 0.05 in Table 2j-WT Diest vs WT Pro of the current study. Similarly, DiCario 2017 identified 26 DEGs at Proestrus vs Estrus in the hippocampus (Table 4). Among the DEGs identified at the same stages Proestrus vs Diestrus in the current study, only 8 of 26 DEGs are occurred in the in Table 2i-WT Pro vs WT Est of current study, 7 of 26 DEGs were not found, and 11 of 26 DEGs in the DiCario’s DEG list are not significant with P> 0.05 in Table 2k-WT Pro vs WT Est of the current study. In addition, there DEGs identified at Proestrus vs Metestrus in the hippocampus by DiCario 2017 (Table 4) do not occur or are not significant in Table 2g WT met vs WT pro of the current study. Another 3 DEGs identified at Estrus vs Metestrus in the hippocampus by DiCario 2017 (Table 4) do not occur or are not significant in Table 2h WT Met vs Est of the current study. The authors should discuss these discrepancies.”

8. Please explain how the data in extended Figure 2-1 were generated.

9. Please include discussion of the following findings by Iqbal 2020: “among 37 DEGs identified at Proestrus vs Estrus, 7 of them were validated by qPCR. Among the DEGs identified at the same stages Proestrus vs Estrus in the current study, only 3 genes occur in the list of 7 validated DEGs.”

10. Validation of a few of the top DEG via qPCR that were identified at the proestrus and estrus stages will increase the reliability of the reported results.

11. Lines 457-458: The following sentence is difficult to understand: “We hypothesized that this could result from subtle KO effects on expression within each genotype group that act oppositely on the sexes.” What is meant by the phrase “act oppositely on the sexes”?

12. The results section pertaining to the DEGs in male and female KO mice should be revised to clearly describe the comparisons. Also, it may be necessary to perform more direct comparisons if they are indeed missing.

a. ‘Are there DEGs in WT estrous stage comparisons that are also DEGs in DO estrous stage comparisons that show opposite directions, so upregulated in one comparison and downregulated in the other? If that is true, the number of genes that change direction could be listed.”

b. Or is it that the gene expression levels are dramatically different between WT and KO as possibly reported in this sentence: “Analysis of genes DE between KO males and a KO estrous stage at FDR < 0.01 showed several sizable groups of genes where *Cnih3* KO.” accentuated a normal sex difference seen in WTs.” This section could be explained better.

13. Please ensure that tabs in the extended table files are fully described.

Minor Concerns:

1. If the mice were used for anything other than the RNASeq analyses, please add this information.

2. Please add more detail to the legends of Figures 6D and 6E. It is important to know what was plotted in each figure.

3. To improve readability, the authors should add a table to highlight a few of the top DEGs discussed in the text. This may help the audience understand the significance of the findings as well as improve readability.

4. The rationale for studying *Cnih3* KO mice should be strengthened. The significance of the findings from the KO mouse experiments should also be discussed.

## Author Response

Synopsis:

The reviewers agree that the authors have addressed an important, and often unexplored topic in neuroscience and biomedical research at large. Using dorsal hippocampal tissue, the authors generated RNA-Seq data to compare males to females from each stage of the estrous cycle and to compare females to each other in C57BL/6J and *Cnih3* KO mice.

The authors report that only 6 genes were significantly differentially expressed between WT male and female animals, while 62 differentially expressed genes (DEGs) were observed between WT males and WT females at specific stages of the estrous cycle. The robust differences (>5000 DEGs) were observed after comparing WT females to WT females in the different stages of the estrous cycle. When comparing the *Cnih3* KO male and female mice, ∼ 850 DEGs were observed; however, comparisons of males to females in specific stages of the estrous cycle produced ∼5,000 DEGs. Additionally, many DEGs were observed between KO females across the estrous cycle.

Given that fluctuating ovarian hormones can have significant effects on neuroplasticity, including human brain disorders, the data produced in this manuscript could provide a vital resource for investigators interested in estrous-specific gene expression patterns in the adult dorsal hippocampus. Also, the interaction between *Cnih3* and the estrous cycle is intriguing and could provide insights into the molecular mechanisms that underlie gene by sex hormone interactions in brain health and disease. However, enthusiasm for this manuscript was significantly reduced due to concerns about the age of mice, tissue collection, RNA extraction, library preparation, and data analysis, which lead to results that are contradictory to what is already available in the literature (which is not well-cited).

Based on the reviewer’s comments*, the current manuscript requires significant editing of all sections (∼2 months or more) before it will be ready for resubmission.

*When appropriate, I have quoted individual reviewers - these are indicated by italicized text.

Major Concerns:

1. As currently written, the background lacks relevant discussion of gene expression and brain structure and behavioral changes across the estrous cycle. The authors should cite the relevant literature, such that the novelty of the authors’ findings are made more clear.

For example:

a. Related to this, the authors should reference the following papers that have compared DEG in females during the diestrus and proestrus phases to males: Thanks for making us aware of these highly relevant studies that were omitted from the original draft. We have provided excerpts of new text integrating these key works. i. one paper has done this in the adult mouse ventral hippocampus (Jaric et al. 2019, Nat Commun; PMID: 31253786)

We have added this source as follows: ‘It has been shown that neuronal chromatin levels and transcriptional activity in the hippocampus fluctuate across the estrous cycle, with measurable effects in behavior and development of psychiatric disease in a sex-specific manner. Specifically, genes involved in synaptic transmission were highly expressed in proestrus female mice compared to diestrus females and males, corresponding to increased synaptic density and number during proestrus (Jaric, 2019).” ii. Duclot and Kabbaj (Genome Biol, 2015; PMID: 26628058) in the rat prefrontal cortex that also explores gene expression differences across the estrous cycle and sex

We have added this source as follows: ‘Additionally, another study found transcriptional differences in genes implicated in sex-specific control of social stress across the estrous cycle that could not solely be explained by sex chromosomes. These changes led to alterations in cellular communication in the female rat medial prefrontal cortex, highlighting the importance of characterizing transcriptional changes across the estrous cycle (Duclot and Kabbaj, 2015).”

b. The authors state “Regular fluctuations in females across the human (menstrual) or rodent (estrous) cycle are known to continually alter the microstructure of the brain.” While this is a true statement, discussion is limited to fluctuations in dendritic spines in rodents. The authors should include discussion of changes in the human hippocampus across the menstrual cycle, including the discoveries of Julia Sacher and colleagues. Thanks for helping us provide an improved broader synopsis of female hormonal cycles and neurobiology. Our current draft expands the presentation substantially by including more diverse sets of both human and mouse examples of such phenomenon in the introduction (some of which is also pertinent to our results regarding cycling gene sets): ‘Regular fluctuations in females across the human (menstrual) or rodent (estrous) cycle are known to continually alter the microstructure of the brain; for example, hippocampal synaptic density peaks when estradiol and progesterone are highest during proestrus (Woolley and McEwen, 1992), which has been shown to affect learning and memory (Frick et al., 2015; Frick and Kim, 2018). A longitudinal study in humans shows rapid microstructural changes in the female hippocampus across the menstrual cycle that suggest increases in myelination during high estrogen periods, paralleling changes seen in rodent models during short-term learning (Barth et al., 2016). Likewise, hormones influence the function of signaling within the brain and between tissues, as estrogens and progestins alter dopamine turnover, suppress GABA signaling (Del Río et al., 2018), and stimulate hypothalamic- pituitary-adrenal (HPA) axis-driven corticosteroid release by upregulating CRH (in contrast to its repression by androgens) (Bao et al., 2006; Lund et al., 2004). Outwardly, the impact of sex has been observed in neuropsychiatric diseases and rodent models thereof, including addiction (Kuhn, 2015), potentially via the hippocampus (Kohtz and Aston-Jones, 2016). It has been shown that neuronal chromatin levels and transcriptional activity in the hippocampus fluctuate across the estrous cycle, with measurable effects in behavior and development of psychiatric disease in a sex-specific manner. Specifically, genes involved in synaptic transmission were highly expressed in proestrus female mice compared to diestrus females and males, corresponding to increased synaptic density and number during proestrus (Jaric et al., 2019).”

c. It is important for the authors to discuss why they focused on the dorsal hippocampus, given the region-specific functionality of the hippocampus as it relates to learning and memory as well as emotion regulation. Thank you for making us aware that we had provided inadequate background. Our revised manuscript now provides key rationale for our having targeted the dorsal hippocampus. We have added the following text to the introduction to clarify why the dorsal hippocampus was our region of focus: ‘Given recent evidence that *Cnih3* is strongly expressed in the dorsal hippocampus and that *Cnih3* KO leads to cellular and synaptic plasticity changes in the dorsal hippocampus of female mice in a cycle-dependent manner (Frye et al., 2021), this study opted to focus its study to the dorsal hippocampus, a region which plays a key role in learning and memory.”

d. Please include more background information on the *Cnih3* KO mouse since these data are central to the manuscript. What is the specific role of this gene? The authors should clarify the possible “buffering effect” that this gene (or better to say its product) may have against sex hormones. Thanks-how this “buffering effect” may be exerted is an extremely important question. We hope that our results will motivate additional research in this important area. We have now laid out a more coherent story for how we came to hypothesize a buffering effect based on behavior, and subsequently tested it in this study. As for particular mechanisms underlying the buffering effect, our discussion extensively covers potential downstream transcription factors and cell types-especially oligodendrocytes-that may be central to this buffering effect. The discussion highlights these genes and cell types, but only implicitly suggested to relate to the buffering effect to avoid being overly speculative (as much additional informatic and experimental work would be required to demonstrate these mechanistic relationships, and our analyses yield a large number of candidates for which this would need to be tested). Given all of this, the discussion now contains the following: ‘[...] All of these results led to the hypothesis that *Cnih3* in some way buffers against hormone-dependent sex differences, with the loss of the protein unmasking deficits in KO females. We therefore directly examined expression differences in WT vs KO altogether and between males or estrous stages. The 514 genes we identified as differentially expressed between genotypes highlighted some shared enrichments with estrous-(dys)regulated gene sets at the level of cell types (SST interneurons, Scng-expressing neurons) and transcriptional regulators (Neurog3); however, most functional enrichments were distinct, spanning several forms of RNA processing and transport, as was the case for candidate upstream regulators, which included Neurod1, Hsp90, and, interestingly, X-binding protein 1 (Xbp1). When we compared single estrous stages or males across genotypes, however, we identified very little differential expression. The combined observation of broader differential expression between male and estrous stages within KOs compared to within WTs, despite the absence of genotype differences, suggested to us that subtle changes were being induced by *Cnih3* KO to accentuate sex-differential expression. Indeed, we observed that the magnitude of sex-differential expression at diestrus or estrus was consistently greater in the KO (Figure 6E), confirming our hypothesis that *Cnih3* buffers against excess gene-regulatory responses to cycling sex hormones.” We also expanded background on the known molecular role of *Cnih3*, mentioning it in the abstract and expanding the description of *Cnih3* in the introduction and in the discussion, such that the introduction also contains the following: ‘Indeed, it was recently observed that knockout (KO) of the opioid dependence-associated gene *Cnih3* (Nelson et al., 2016) has not only sex-, but estrous stage-specific effects on learning and memory (Frye et al., 2021), supporting a role for acute sex hormonal effects on addiction-relevant brain circuitry related to learning and memory. *Cnih3* is an AMPA receptor trafficking protein that delivers AMPARs to the postsynaptic membrane and thus enhances its activity (Schwenk et al., 2009; Herring et al., 2013; Shanks et al., 2014). AMPA receptors in the hippocampus have a clear role in learning and memory (Lee et al., 2003; Kessels and Malinow, 2009). Indeed, consistent with *Cnih3* modulating learning, KO showed severe memory deficits, altered biochemical localization of AMPA receptors, and corresponding synaptic changes, but only in female mice, and limited to particular phases of the estrous cycle (Frye et al., 2021). This suggested the surprising hypothesis that *Cnih3* is involved in buffering the female brain against effects of hormones on hippocampal learning, and thus loss of *Cnih3* results in these abnormal outcomes in a cycle-dependent manner.”

2. The authors identified DEGs in females across the estrous cycle and compared their findings to those previously observed in C57BL/6 mice. While the authors suggest agreement of the datasets, there is no mention of the prior finding of only 82 DEGs in the hippocampus across estrous cycle stages, with fewer in other brain regions. With the authors findings of many DEGs in females across the estrous cycle, it is important for the authors to confirm these findings considering that females are increasingly included in studies of biomedical research. Thank you for drawing this important contrast between our work and this prior study. While the imposition of a fold-change cutoff is not explicitly mentioned anywhere in the Dicarlo paper, analysis of their provided mean FPKMs by gene in each stage and region shows that their DEG calls were fold changes of 1.5-fold or greater. By contrast, we called DEGs based on FDR alone regardless of fold changes. Thus, just this choice of cutoff likely explains the difference between the two papers. Indeed, if we were to limit to only the high magnitude (>1.5 changes) we find 297 unique autosomal genes (and an additional 10 sex chromosomal genes with differential expression (511 pairwise autosomal DE events and 22 sex chromosomal DE events). We have incorporated discussion of this difference into our limitations paragraph of the discussion quoted in our response to 3a below.

3. Many methodological concerns need to be addressed: a. The assessment of estrous cycle phase is not ideal. Typically, the mouse would be followed for a couple of complete cycles, assessing phase daily, to ensure that the mouse is having regular cycles. Given that the heat map in Figure 2E depicts variation within the diestrus group, this could be contributed to how long the diestrus phase lasts (∼48h) along with the fact that mice in early diestrus will look hormonally and molecularly different from those in late diestrus. The authors should address this possible issue of staging animals for 2 days only and how this methodological approach could have impacted their data (e.g., early vs late diestrus and variability in your data). Thank you, we were unaware that a subset of past estrous-centered studies have used such rigorous staging protocols! Toward both that limitation and others (e.g., diestrus subclustering), we have added a paragraph to the discussion section covering limitations, which we unfortunately did not include in our original draft. That new text also follows for convenience: ‘Our study has several limitations. Some previous studies examining estrous stage and hippocampal biology have performed extensive estrous staging across multiple cycles (Jaric 2019, Wooley and McEwen 1992), whereas here, we only performed cytologic staging on two consecutive days (the day before tissue collection and at tissue collection), which could potentially lead to inclusion of brains from mice with irregular cycling. Additionally, the cytologic diestrus phase consists of endocrinologically distinct early (low estrogen) and late (rising estrogen) phases. Our sample clustering (Figure 2E) suggests that a portion of our WT diestrus mice may have been split between two transcriptional states, which most likely represented these endocrine subphases. We note, however, that the two subclusters of WT diestrus samples are similarly sized, such that our overall data for WT at diestrus should represent the composite cytologic stage. Finally, we were deliberately somewhat liberal in our thresholds for calling DEGs (only requiring an FDR<0.05 without an additional fold-change cutoff). While this procedure will result in detection of some smaller magnitude changes at the level of single genes, it aids our gestalt analysis by casting a broader net for genes subject to any degree of fluctuation across the estrous cycle.”

b. The 3-6 month age range in the mice is large when comparing gene expression; studies demonstrate significant DEG in mice at different ages. Including a list of the age of each mouse at the time of sacrifice will help address this issue. Thank you for suggesting this important addition, which is now part of

Supplementary Table 1.

c. Please add more detail to the dissection of the dorsal hippocampus (e.g., what landmarks were used to determine uniformity?; were all mice dissected at the same time of day and by the same person?). Thank you for making us aware of these oversights including consistent sampling time and personnel which help highlight the rigorous methods of our RNA-seq experiments. We have added this key information to the methods section as quoted in response 3E below. (Briefly, all mice were dissected in the afternoon by the same researcher using methods previously described (Xia et al., 2011), with the dorsal-most third of the hippocampal formation being isolated for downstream RNA-seq.)

d. Please be clear that the data are generated from the dorsal hippocampus. Throughout the manuscript, it often appears that the entire hippocampus was used for the analysis. Note that the estrous cycle data on the ventral hippocampus is available (Jaric et al. 2019; PMID: 31253786).

Thanks for the suggestion. We have added “dorsal” to the title of the paper as well as to several topic/concluding sentences which has improved the clarity of our descriptions.

e. Please provide more detail surrounding the lysis of the hippocampus, including the use of homogenization.

We have added text to the methods section to clarify how homogenization was performed: ‘Dissected dorsal hippocampi were placed in 500 μL of buffer (50 mM Tris, pH 7.4, 100 mM NaCl, 1% NP-40, supplemented with RNAsin and protease inhibitors) on ice. Samples were homogenized in buffer solution on ice using a handheld motorized pellet pestle, and lysate was then centrifuged at 2,000 x g for 15 minutes at 4{degree sign}C, and 133 μL of the supernatant was taken forward for RNA purification. This was mixed with 67 μL of Promega’s simplyRNA Tissue Kit Homogenization buffer with 1-thioglycerol (20 μL per mL), then 200 μL of Promega’s lysis, and extracted using a Maxwell RSC 48 robot (Promega) following the manufacturer’s instructions for the above Kit.”

f. The use of a small amount of RNA (10ng) for library prep should be explained, since this was bulk tissue from which much more RNA can be recovered. Please list the average weight of hippocampal tissue used to isolate RNA and explain why only 10 ng of RNA was used for library prep when hundreds of ngs of RNA could be available to generate high quality, high diversity libraries with low PCR cycle number? Relatedly, please answer these questions: We again appreciate the reviewers’ comments that have helped us provide important methodologic detail throughout the manuscript. We have addressed each point briefly below.

Regarding tissue weights, we did not weigh the hippocampal fragments and therefore cannot provide the weights of the tissue itself. However, we do have records of RNA yield and RINs for all samples (now included in Supplemental Table 1). Our RNA yields from these extractions were indeed in the 100’s of ngs total (396-870ng in 30 uls).

Our local sequencing core provides a range of options for library prep depending on the amount of input material-but as our lowest-concentration samples were just below the recommended minimum for their ’ribodepletion’ strategy, they recommended (and we agreed to) using the Clontech SMARTer kit. It is a product we are familiar with for both low (<50ng sample) and high (>500ng per sample) input applications, and which we have reliably seen high quality and high diversity libraries from 10ngs of total RNA. This is borne out in the alignment QC metrics that we did not initially share, have likewise now added to Supplemental Table 1. See columns on % uniquely mapping reads (generally {greater than or equal to} 90%), etc.

i. Was RNA polyA enriched? Yes, thanks for suggesting that we state this explicitly. The Clontech SMARTer ultra low input kit is a highly regarded commercially available RNA library kit that utilizes pseudo-random oligo-dT primers and it does preferentially target full length poly-A tailed mRNA. Please see Clontech (https://www.takarabio.com/resourcedocument/x33064) for more details. We have added this detail to the methods text.

ii. Can you show the number of sequencing reads per animal/biological replicate and better explain how different replicates were considered? Thank you for making us aware of this important oversight. It is now

a column in Supplementary Table 1. Regarding definition of replicates, we understand and apologize for technical issues with the transmission of figures and legends that made this unclear. However, in the Figure 1 legend, which we hope the reviewers can now see as intended, we state that “Dorsal hippocampi from male and female adult C57/B6 wild-type (WT) and *Cnih3* knockout (KO) mice were collected for RNA-sequencing (one hippocampus/mouse = one sample).”

iii. Can you discuss why you did not use Deseq2 for gene expression analysis?

Thank you for your helpful suggestions that have helped us improved our description of our analytic strategy. DESeq2 is one of the three most popular methods (EdgeR, DESeq2, and Limma with Voom) for RNA-seq differential expression analysis where the popularity of DESeq2 is considered the easiest to use at the expense of the limited ability to model sample and gene specific variances, especially those that differ from the expected negative binomial distribution. This leads to higher numbers of false positives, a problem illustrated by Li, et. al in Genome Biology (https://doi.org/10.1186/s13059-022-02648-4). The Limma with Voom method we employed here was shown to be more reliable and allowed for the use of contrast matrices, which negated the need for post-hoc multiple testing corrections.

iv. How did you account for the use of pairwise comparisons in your analysis?

Thank you for making us aware that this was not clearly described.

As alluded to above, the use of contrast matrices with Limma and Voom directly incorporates multiple testing corrections within the Benjamini-Hochberg p-value adjustments for all contrasts coming from the same statistical model. It is not necessary to perform post-hoc p-value adjustments on 2 or more comparisons from a single contrast matrix (i.e., downstream of a joint model run on the entirety of the samples), whereas post-hoc adjustments would be necessary if you were to perform 2 or more independent pairwise comparisons (e.g., subsetting the data to two conditions at a time and running individual models for each pair of conditions in that subset) with something like DESeq2. We have added a similar explanation to the methods text: “All differential expression analyses used a single input dataset covering all WT and *Cnih3* KO samples and model using a Limma contrast matrix to avoid the need for post-hoc multiple testing corrections: expression ∼ 0 + group (where group = genotype and estrous stage or male).”

g. Please clarify why the library was amplified 12-15 cycles. Specifically, what determined the number of cycles, if all the samples started with equal amounts of RNA. Thanks for catching this. We have verified with the sequencing core, and clarified in the text, that the specific number of cycles for all samples was 14. (The “12-15 cycles’ language came from their internal methods’ generic text). Therefore, all samples started with equal amounts of RNA and proceeded through identical sequencing preparation reactions as would be expected.

h. It is unclear if all the samples were included in the differential expression analysis. The manuscript would benefit from the inclusion of PCA plots of the RNA-Seq dataset (at least one for WT and KO separated) that would demonstrate how the difference groups segregate. This is particularly useful when considering only 6 DEGs were found between WT males and females but thousands were identified between WT females at different stages of the estrous cycle from an inbred mouse line. Since the heatmap in Fig 2E suggests that some mice have similar gene expression profiles, a PCA plot would further demonstrate how different each group is from another.

Thank you for this excellent suggestion. We have added two PCA plots (with and without sex chromosomal genes considered), the new Supplementary Figures S4 and S5, to the current manuscript.

4. Please include a table that lists the DEGs from each comparison, in addition to the inclusion of the VENN diagrams.

Thank you for pointing this out. While Supplementary Table 4 does have the DEGs for each comparison, with each comparison as a separate sheet, we have now highlighted rows indicating the genes and Enrichr results we considered significant using our criteria as defined in the text. In addition, readers who would like to filter for just the genes at a certain cutoff can use Excel’s filter function to filter to any cutoff nominal p-value, FDR, and/or logFC threshold they desire. Additionally, the Supplemental Tables now have highlighted rows indicating the genes and Enrichr results we considered significant using our criteria as defined in the text.

Table 4, tab 4l in the supplemental material should be added to the main text, with the inclusion of a legend.

We agree that this is a useful summary of the first two sets of analyses and helps visually close out the within-genotype portion of the manuscript. Table S4L is now part of Figure 4. 5. Please revise the last paragraph of the Results section. It is hard to decipher as currently written and would be easier to follow if the comparisons were described more thoroughly (e.g., referencing earlier figures when talking about which groups are being compared). For example, from line 336: “small changes occur within multiple subgroups of KO relative to their sex/estrous matched WT counterparts”. Explaining which subgroups of KO and WT counterparts are being described would be useful.

As suggested, we have revised the end of the results section to hopefully read more clearly . The results section now ends as follows: ‘The findings thus far are surprising when considered in conjunction.

Specifically, how is it that estrous stage-to-male comparisons within KO groups reveal substantially more DEGs than the cognate comparisons in WT (Supplementary Table 4l), while comparison of a given stage between KO and WT revealed very few DEGs (Figure 6C). We hypothesized that this could result from subtle KO effects on expression within each genotype group that act in oppositely on the sexes. Such effect patterns would be consistent with both the paucity of cross-genotype DEGs and the increased number of DEGs seen between KO males and KO estrous stages relative to the same comparison within WT groups.

To examine whether this hypothesis was valid at the level of KO male-vs-stage DEG sets, we again utilized the degPattern algorithm, now with the intention of visualizing the changes in WT expression patterns of those genes identified as

DE in KO male-estrous stage comparisons. Consistent with our hypothesiswe see that DEGs are generally altered in opposite directions in male and female KOs relative to their WT peers (shown are the two KO estrous stage-male comparisons with the most DEGs). The overall conclusion is that KOs have accentuated sex differences across large portions of the dorsal hippocampal transcriptome (Figure 6D, Supplemental Tables 6A-6B). Enrichr analyses of genes following each pattern for these two male-stage comparisons (such as M>diestrus in WT with male upregulation and female downregulation in KO) are provided in Supplemental Table 6C, and recurring terms across genes with different patterns are tabulated in Supplemental Table 6D. To replicate this observation of accentuated sex differences in the KOs, we also examined all genes differentially expressed between males and diestrus or between males and estrus, and plotted their absolute log2 fold change values for both WT or for KO mice (Figure 6E). At any FDR thresholds tested, a non-parametric Wilcoxon test identified extremely significant increase in the magnitude of the absolute sex differences in the KO compared to those seen in WT. This confirms the obsevation of a general net increase in sex-differential expression between *Cnih3* KO males and KO females during diestrus and estrus.”

6. Please discuss why the gene cluster analysis was preferred over analyses such as GO or pathway analyses.

We apologize for the fact that we did not make clear that the Enrichr package we used is a tool that examines gene set databases including the canonical ones such as GO, KEGG, etc. Indeed, several of the significant categories presented are from GO. It is simply that to create those gene sets for GO pathway analysis, we used a cluster analysis to identify the DEGs that shared the same pattern across the cycle. We then perform Enrichr analysis on the groups of genes that cycle together.

The advantage of this approach was it could identify pathways that correspond to specific patterns of gene expression fluctuation across the estrous cycle and thus likely correspond to specific endocrine/neuroendocrine stimuli. We thought this approach is thus more biologically related to cyclic expression patterns, and more interpretable than simply running enrichment analysis on all genes differentially expressed between two given stages.

To make this workflow more clear, we have added a new panel to Figure 1 that shows the analytical workflow and illustrates the types of gene sets Enrichr tests; we call this figure panel out where we first mention Enrichr in the manuscript.

7. Please clarify the following sentences and make it clear what the rationale was for the analysis/discussion:

a. “Finally, the genes sharply peaking in diestrus of cluster 4 were strongly enriched for Sncg+ neurons and hippocampal CA3 neurons from Allen Brain Atlas single cell RNA-seq, and were enriched for 239 genes upregulated by knockdown of RELA, Neurod1, or Mecp2, or by overexpression of Neurog3.” We have rephrased this sentence and expanded on the rationale for highlighting these results and our interpretation of them: ‘Finally, cluster 4, representing genes sharply peaking in diestrus, was strongly enriched for Sncg+ neurons and hippocampal CA3 neurons from Allen Brain Atlas single-cell RNA-seq. Cluster 4 was also enriched for genes upregulated by knockdown of RELA, Neurod1, or Mecp2, or by overexpression of Neurog3 (Supplementary Table S3b). As some of these TFs are known regulators of neuronal gene expression, this suggests neuronal gene expression is disproportionately altered in this cyclic manner. It is also reassuring that the pathway analyses are highlighting CNS pathways and cell types, rather than other tissues, consistent with a bona fide estrous cycle impact on the brain.”

b. “Overall, driven by the following considerations A) sex differences in psychiatric disorders, including addiction; B) the role of sex hormones in regulating the HPA axis; C) the role of stress in drug reinstatement (Sherry A. McKee, 2015); D) the molecular and behavioral integration of both sex and stress hormone signals in the hippocampus (Bao et al., 2006; Frick et al., 2015b; Joshua A Gordon, 363 2016); E) the prior work showing the influence of estrous stage on hippocampal physiology and spatial learning (Frye, 1995; Warren and Juraska, 1997); and F) the sex and estrous specific differences in hippocampal phenotypes in *Cnih3* KO females, we conducted a well powered study to understand the transcriptional effects of estrous on dorsal hippocampal gene expression.” Thanks- We’ve overhauled this into a structured paragraph: ‘We had a variety of motivations to specifically conduct a study of the consequences of estrous on gene expression in the dorsal hippocampus of both WT and *Cnih3* KO mice. First, *Cnih3* mice are of interest because of the association of CNIH3 to addiciton phenotypes in humans (Nelson et al., 2016), and the initial characterization of these KOs showed a strong interaction between sex and genetics in hippocampal phenotypes (Frye et al., 2021). Second, the dorsal hippocampus is essential for addiction learning (Fakira et al., 2016), and sits at the interface of stress, sex and addiction learning (Henderson et al., 2015; Johnson et al., 2021). This is interesting in light of the role of sex hormones in regulating the HPA axis (Lund et al., 2004), the role of stress in drug reinstatement (McKee et al., 2015), and the molecular and behavioral integration of both sex and stress hormone signals in the hippocampus (Bao et al., 2006; Frick et al., 2015; Padilla-Coreano et al., 2016). Finally, there is also prior work showing the influence of estrous stage on both hippocampal physiology and spatial learning (Frye, 1995; Warren and Juraska, 1997), again indicating the importance of the influence of hormones on this structure, and from thence hormonal behavior.”

8. In the last part of the results section, many comparisons between groups are made but the reason for the analyses are not clear. Thanks-we did not textually convey the transition into a different set of comparisons here. We have changed the leading sentence of this section to make clear that the WT-vs-KO analyses are distinct and across genotype, as opposed to the preceding analyses within genotype: “Subsequently, we performed within-sex/estrous stage comparisons of the two genotypes to better elucidate the effects of *Cnih3* knockout.”

9. The data are overinterpreted in the Discussion section of the manuscript. Given the lack of clarity in the Results section, there is a great deal of speculation within the Discussion section. Additionally, the use of citations should be increased (e.g., 26 references is quite low). We have worked hard to clarify the results section, which we believe makes the discussion better grounded. We deeply edited and expanded the discussion to hew more closely to a description of our results and decreasing speculation (see for example, paragraph 3 of discussion, starting “We then examined data from *Cnih3* knockouts in the same manner, identifying a much more marked extent...”). Additionally, we have added citations and summarized their findings to help contextualize highlighted results, such as enrichment-implicated transcription factors. However, we do still indicate where interesting hypotheses emerge from our analysis for future study, as we feel this is an appropriate use of a discussion section.

Finally, we have increased our referencing throughout, especially by citing the studies that motivated our own, and to better place our results in the context of what has come before.

Minor Concerns:

1. Please ensure that the Supplemental Tables are fully readable when uploaded in the revised manuscript.

We’re very sorry that these key components were inaccessible to some reviewers. We are unsure of what was creating this trouble as far as supplementary tables, as they are all simply multi-sheet excel files (even if admittedly somewhat large).

We have not been able to replicate readability issues of these in-house on Mac or PC.

Please let us know if this problem persists for reviewers. The other possibility (we don’t know at the time of writing) is that they converted to PDF by eNeuro? We hope not, and will work with editors to make sure that native excel versions are also available to reviewers. PDFs of big tables are indeed irksome.

2. Please improve the resolution of Figures 3 and 4 so that it can be interpreted.

Our apologies-we discovered in the course of revising this work that we had not appropriately rendered the figures for the original submission. They should now be both openable and high-resolution.

3. The term used in the manuscript title: “across estrous” does not mean anything; it should be “across the estrous cycle”.

We have re-titled the manuscript in response to the reviewers’ helpful suggestions
