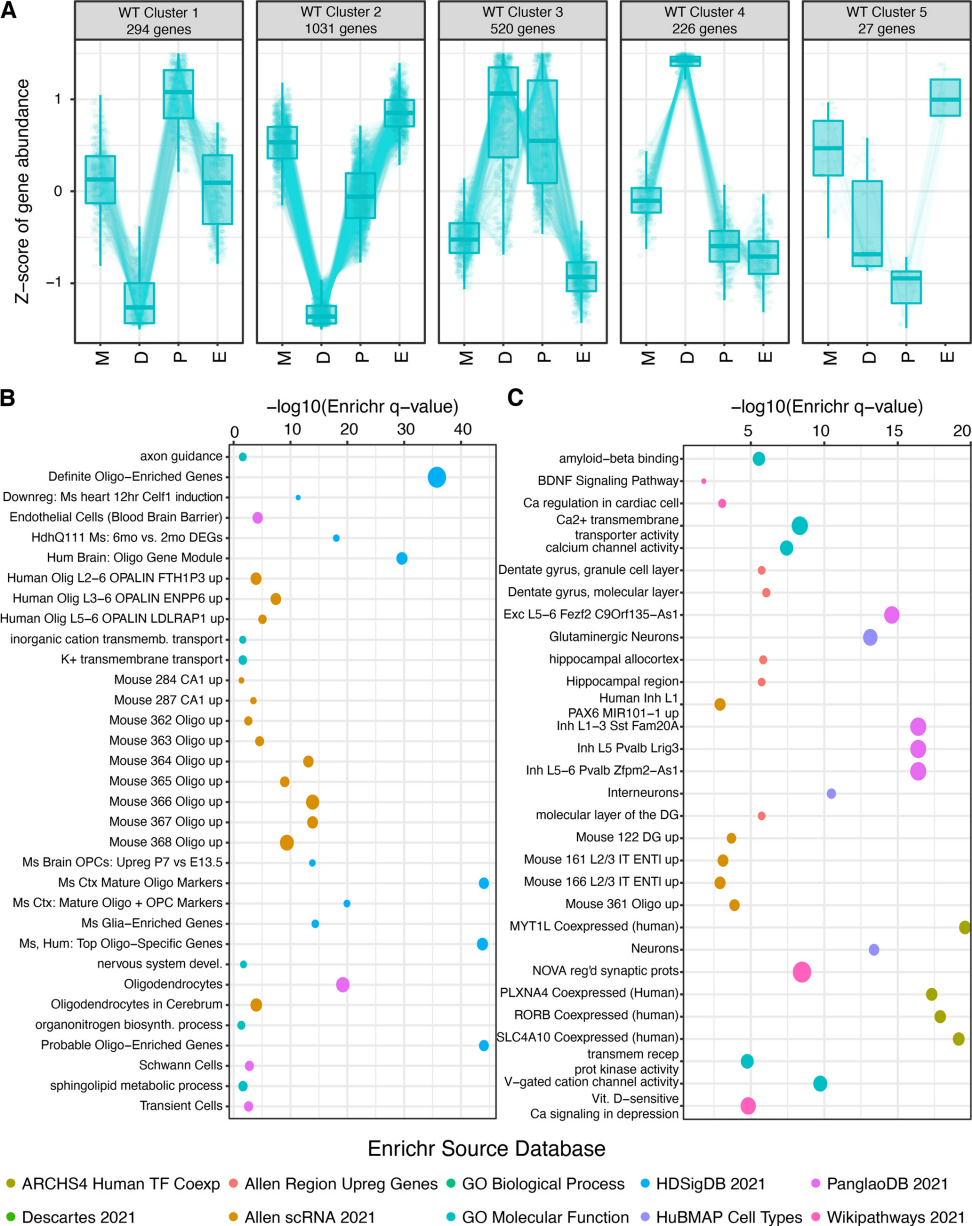# Erratum: Mulvey et al., “*Cnih3* Deletion Dysregulates Dorsal Hippocampal Transcription across the Estrous Cycle”

**DOI:** 10.1523/ENEURO.0155-23.2023

**Published:** 2023-06-01

In the article, “*Cnih3* Deletion Dysregulates Dorsal Hippocampal Transcription across the Estrous Cycle,” by Bernard Mulvey, Hannah E. Frye, Tania Lintz, Stuart Fass, Eric Tycksen, Elliot C. Nelson, Jose A. Morón, and Joseph D. Dougherty, which was published online on February 27, 2023, Figure 3 appeared incorrectly. The points in panel *B* did not match with the corresponding key. The corrected figure appears below, and the online version of the article has been corrected.

**Figure 3:**