# Supplementary figures and images for: Cnih3 Deletion Dysregulates Dorsal Hippocampal Transcription across the Estrous Cycle
Source: eNeuro. 2023 Mar 13;10(3):ENEURO.0153-22.2023. doi: 10.1523/ENEURO.0153-22.2023 (PMC10027183; doi:10.1523/ENEURO.0153-22.2023)

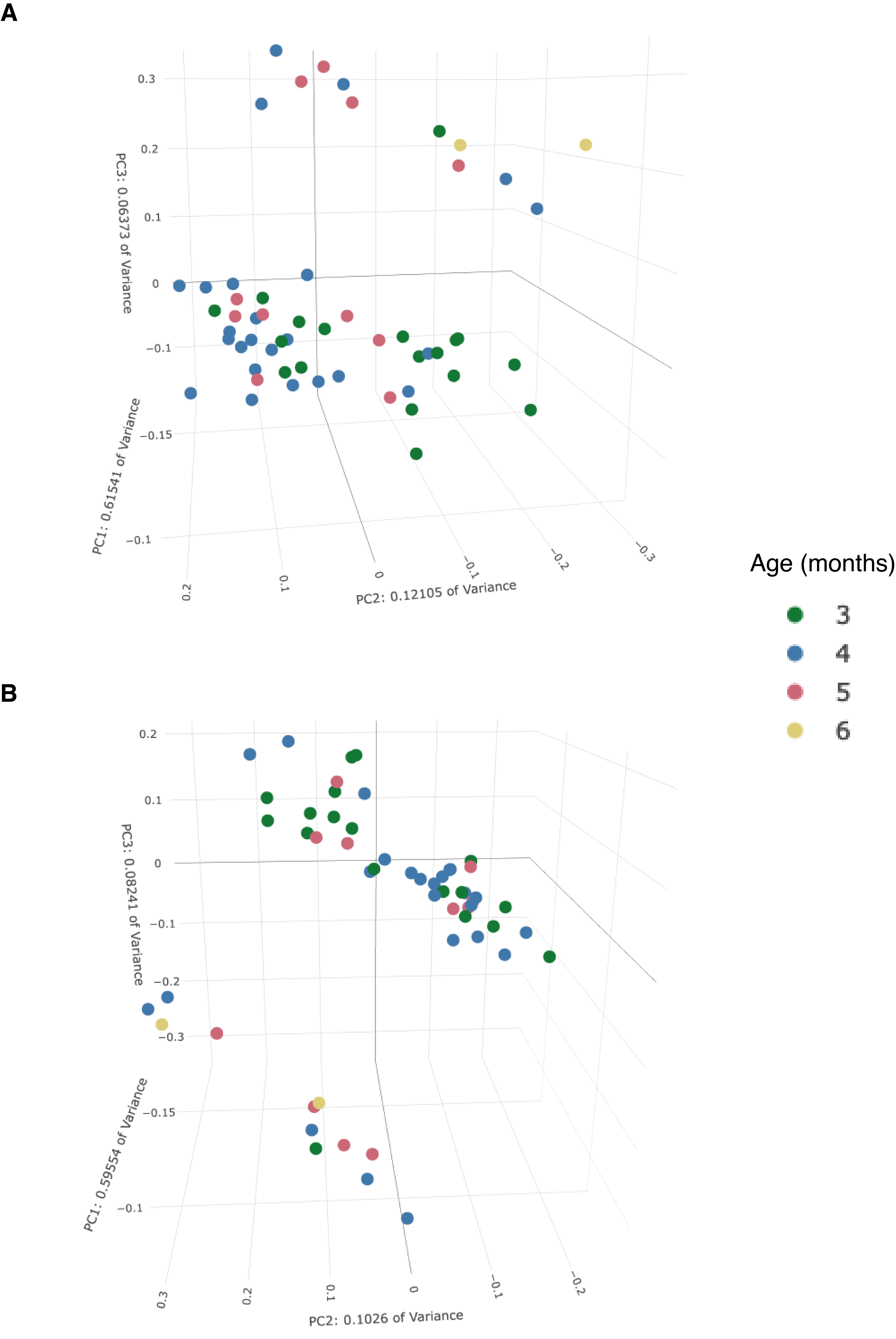

Supplement: Extended Data Figure 1-1 — 3D principal component analysis (PCA) of highly variable gene expression data analyzed with versus without removal of age effects by the ComBat-seq package. Expression values were filtered to those with a standard deviation of ≥1 for PCA calculation and plotting. Similar perspectives shown of 3D PCA of the cohort gene expression values as (A) modeled by limma/voom and used throughout the paper and (B) as corrected for categorical age by ComBat-seq prior to modeling in limma/voom. Download Figure 1-1, TIF file. [file enu-eN-NWR-0153-22-s02.tif]

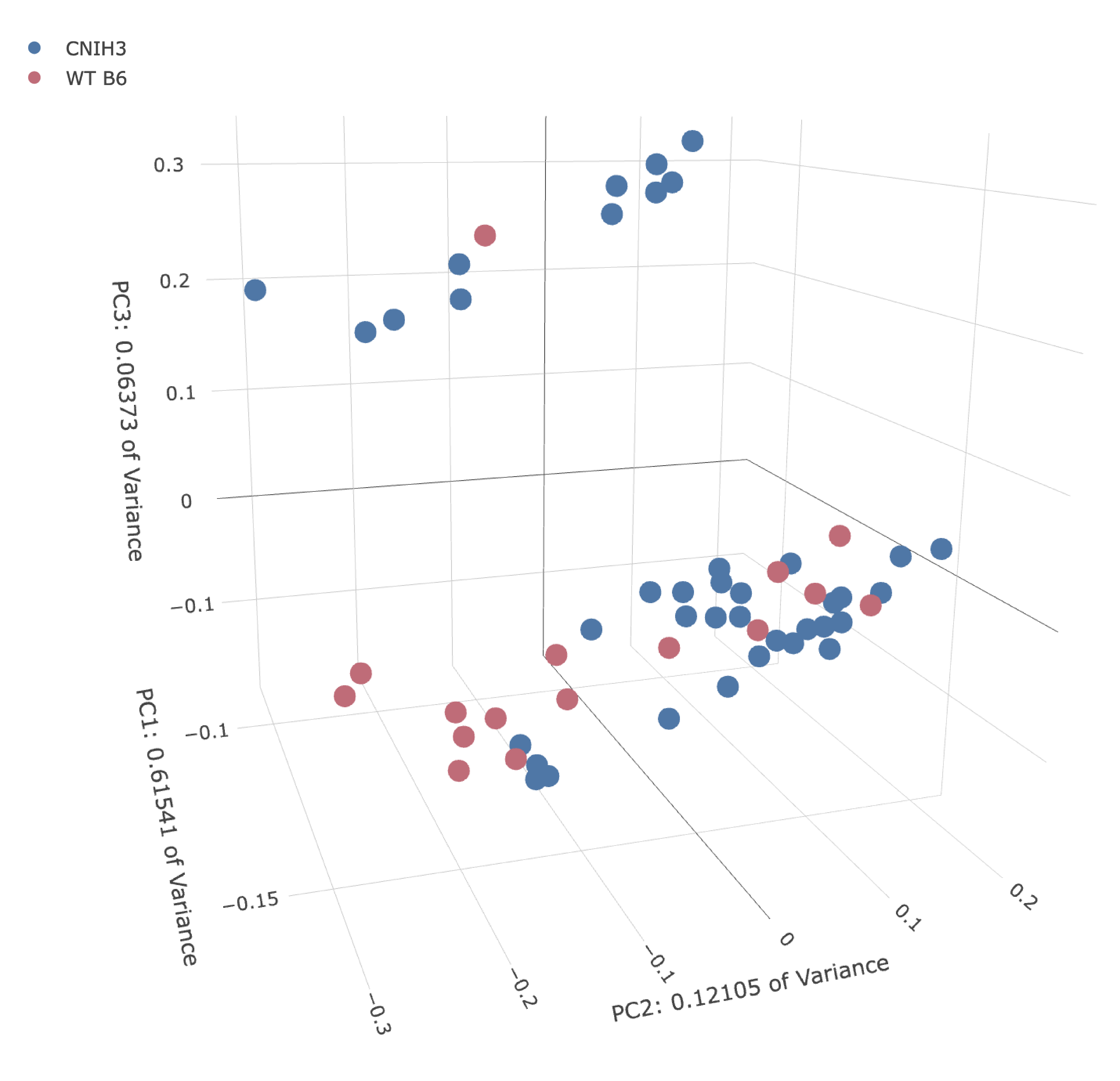

Supplement: Extended Data Figure 1-2 — 3D PCA of highly variable WT gene expression reveals no role of parental genotype in observed DE patterns. Expression values were filtered to those with a standard deviation of ≥1 for PCA calculation and plotting of WT samples. CNIH3: one parent was heterozygous for the Cnih3 mutation (and offspring was mutation-negative); WT B6: both parents were vendor-purchased B6 or their in-house, inbred descendants. Download Figure 1-2, TIF file. [file enu-eN-NWR-0153-22-s03.tif]

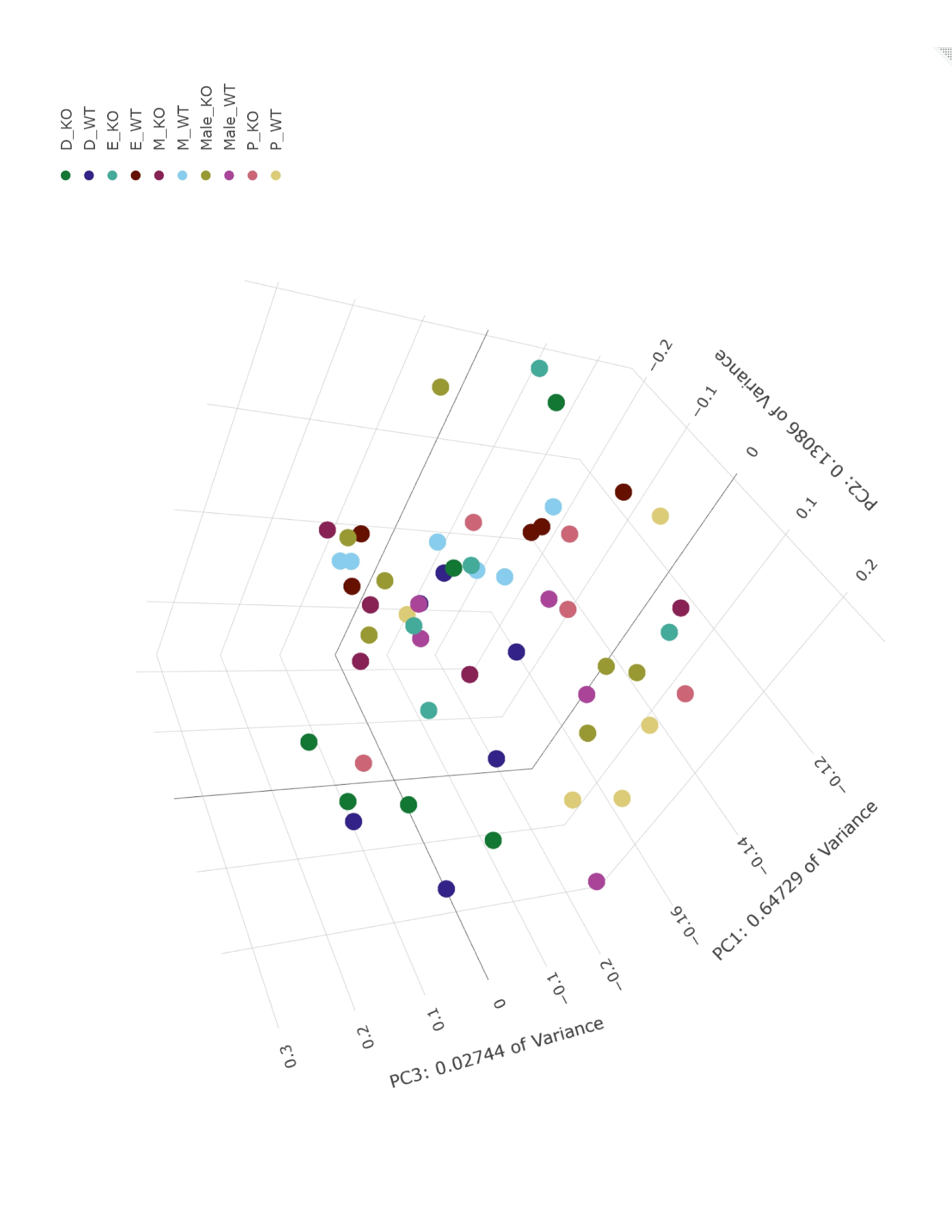

Supplement: Extended Data Figure 2-3 — Three-dimensional PCA of all analyzed samples using variably expressed autosomal genes. Download Figure 2-3, TIF file. [file enu-eN-NWR-0153-22-s06.tif]

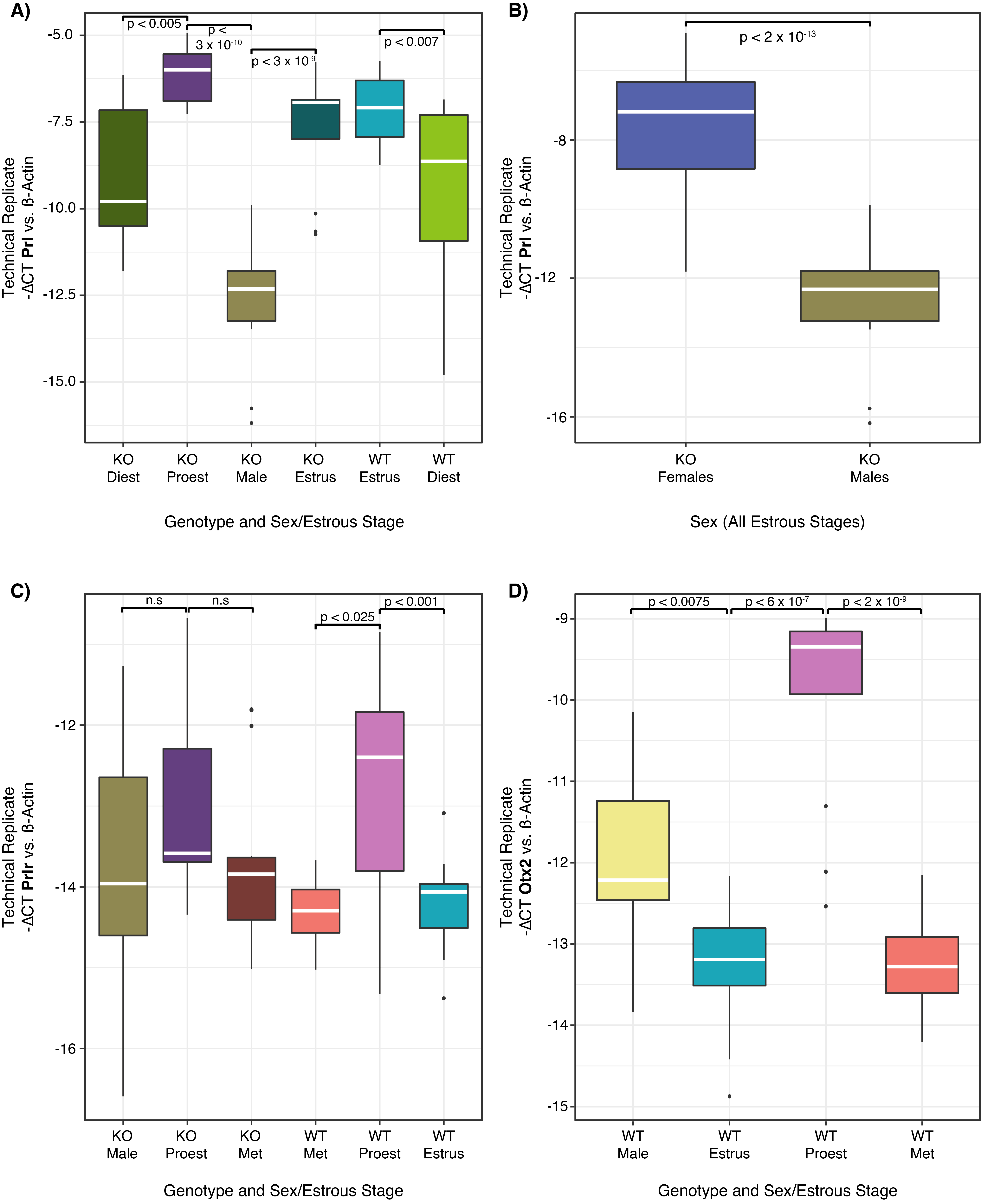

Supplement: Extended Data Figure 2-4 — qPCR validation of three DEGs (Otx2, Prlr, Prl) between estrous stages. Bonferroni corrected p-values (correcting for twelve total comparisons across the three genes) are shown for each comparison preselected for replication by qPCR. Download Figure 2-4, TIF file. [file enu-eN-NWR-0153-22-s07.tif]

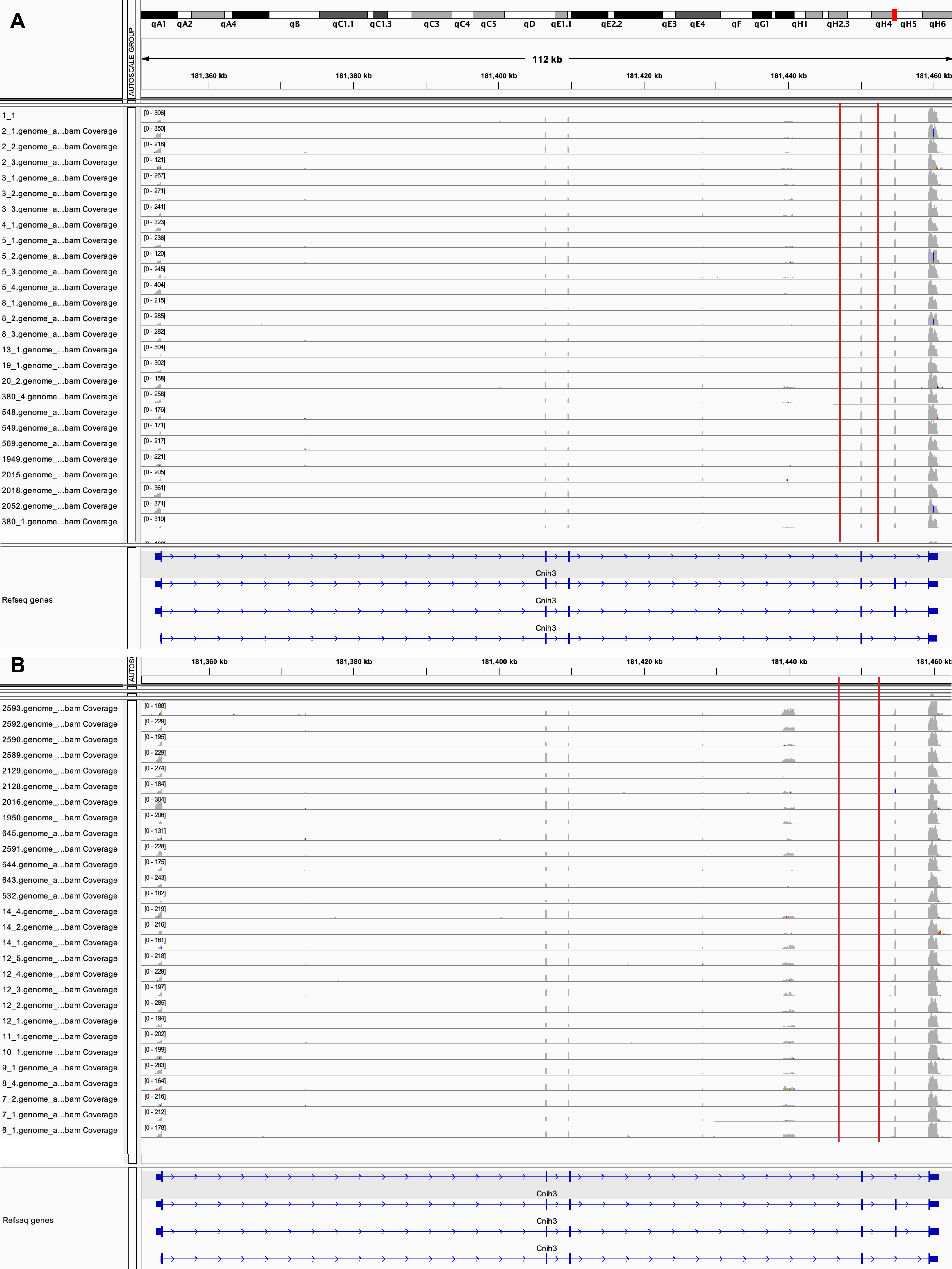

Supplement: Extended Data Figure 4-1 — Cnih3 RNA-seq read coverage in each analyzed sample. A, Coverage in WT samples. B, Coverage in KO samples. Download Figure 4-1, TIF file. [file enu-eN-NWR-0153-22-s08.tif]

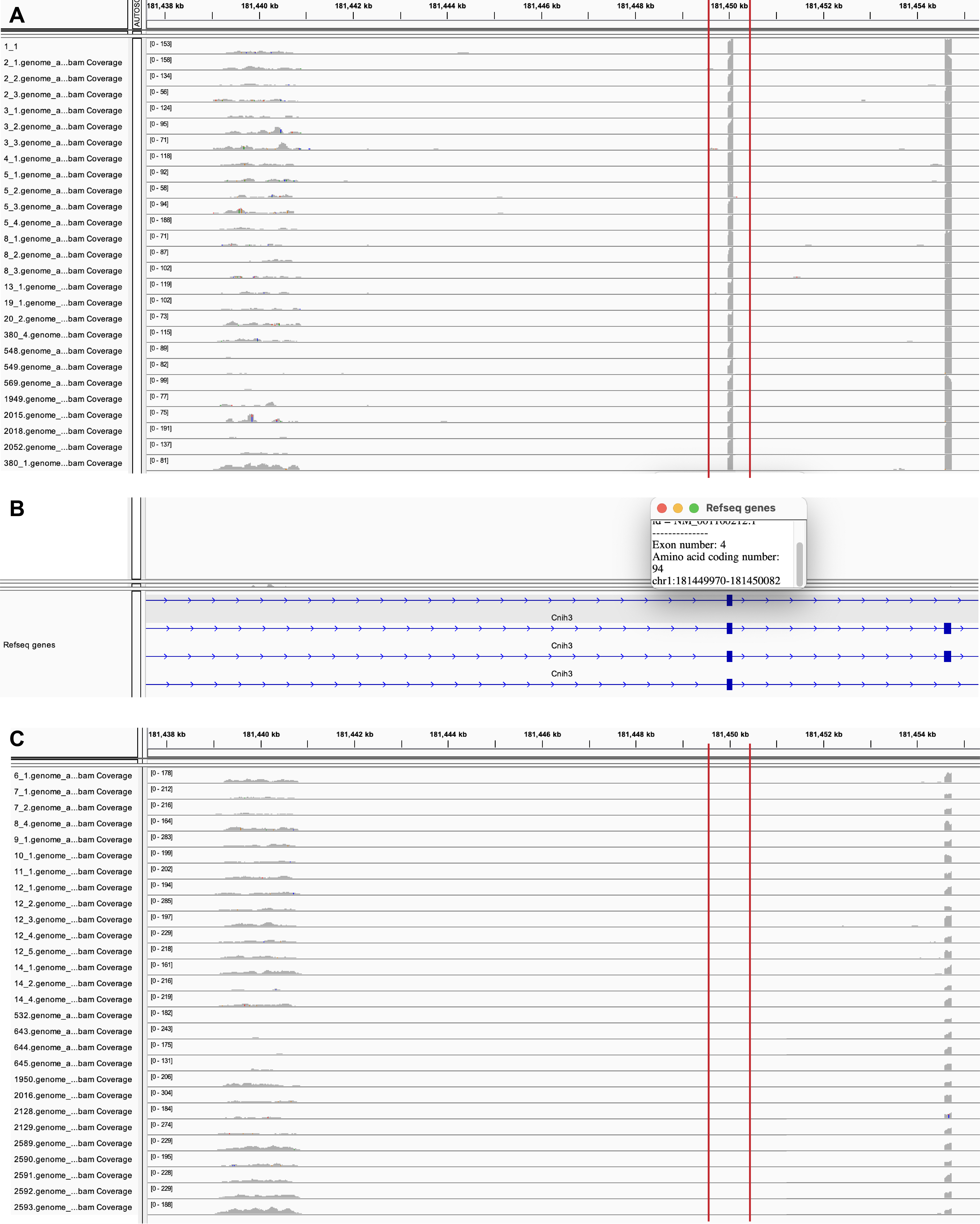

Supplement: Extended Data Figure 4-2 — Cnih3 read coverage recapitulates the KO strain loss of exon 4 previously reported. Higher zoom of the exon 4 region of Cnih3 is shown for (A) WT samples and (C) KO samples, with the Cnih3 gene track and corresponding information on exon 4 shown in between (B). Download Figure 4-2, TIF file. [file enu-eN-NWR-0153-22-s09.tif]

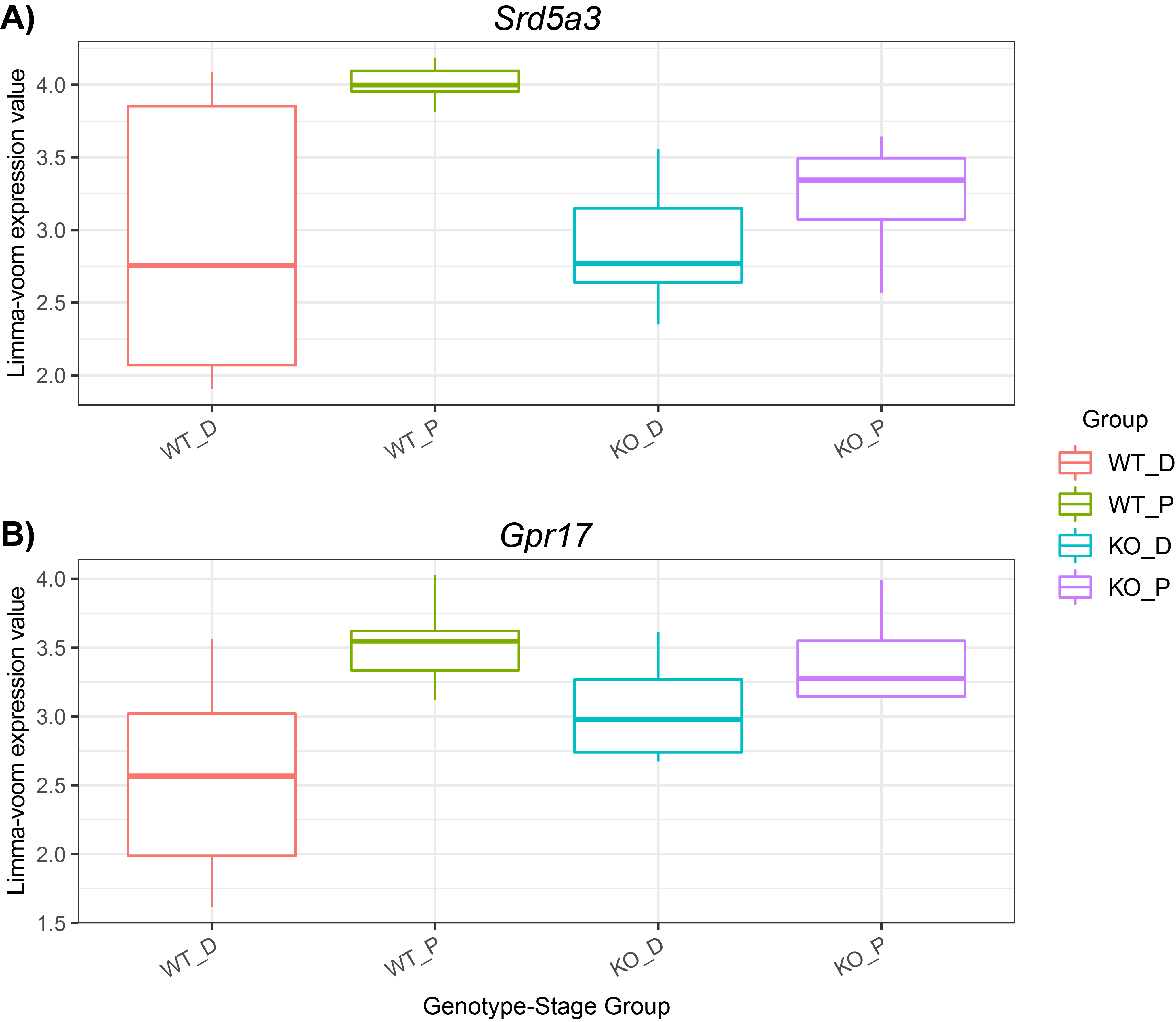

Supplement: Extended Data Figure 5-2 — Example oligodendrocyte-enriched genes found in estrous pattern subclusters showing upregulation from diestrus to proestrus in WT and attenuated proestrus upregulation in KO. A, Srd5a3. B, Gpr17. Di = diestrus; Pro = proestrus. Download Figure 5-2, TIF file. [file enu-eN-NWR-0153-22-s11.tif]
